# Assessment of facility readiness and provider preparedness for dealing with postpartum haemorrhage and pre-eclampsia/eclampsia in public and private health facilities of northern Karnataka, India: a cross-sectional study

**DOI:** 10.1186/1471-2393-14-304

**Published:** 2014-09-04

**Authors:** Krishnamurthy Jayanna, Prem Mony, Ramesh BM, Annamma Thomas, Ajay Gaikwad, Mohan HL, James F Blanchard, Stephen Moses, Lisa Avery

**Affiliations:** Karnataka Health Promotion Trust, IT Park, 5th floor, No 1-4, Rajajinagar Industrial Area, Behind KSSIDC Administrative Office, Rajajinagar, Bangalore 560044 India; Department of Community Health Sciences, University of Manitoba, S113-750 Bannatyne Avenue, Winnipeg, MB R3E 0W3 Canada; Depart of Epidemiology, St John’s Research Institute, St John’s National Academy of Health Sciences, Bangalore, India; Depart of Obstetrics, St John’s Medical College and Hospital, St John’s National Academy of Health Sciences, Bangalore, India

**Keywords:** Postpartum haemorrhage, Pre-eclampsia, Eclampsia, Facility readiness, Provider preparedness, Quality, Maternal care, Public sector, Private sector

## Abstract

**Background:**

The maternal mortality ratio in India has been declining over the past decade, but remains unacceptably high at 212 per 100,000 live births. Postpartum haemorrhage (PPH) and pre- eclampsia/eclampsia contribute to 40% of all maternal deaths. We assessed facility readiness and provider preparedness to deal with these two maternal complications in public and private health facilities of northern Karnataka state, south India.

**Methods:**

We undertook a cross-sectional study of 131 primary health centres (PHCs) and 148 higher referral facilities (74 public and 74 private) in eight districts of the region. Facility infrastructure and providers’ knowledge related to screening and management of complications were assessed using facility checklists and test cases, respectively. We also attempted an audit of case sheets to assess provider practice in the management of complications. Chi square tests were used for comparing proportions.

**Results:**

84.5% and 62.9% of all facilities had atleast one doctor and three nurses, respectively; only 13% of higher facilities had specialists. Magnesium sulphate, the drug of choice to control convulsions in eclampsia was available in 18% of PHCs, 48% of higher public facilities and 70% of private facilities. In response to the test case on eclampsia, 54.1% and 65.1% of providers would administer anti-hypertensives and magnesium sulphate, respectively; 24% would administer oxygen and only 18% would monitor for magnesium sulphate toxicity. For the test case on PPH, only 37.7% of the providers would assess for uterine tone, and 40% correctly defined early PPH. Specialists were better informed than the other cadres, and the differences were statistically significant. We experienced generally poor response rates for audits due to non-availability and non-maintenance of case sheets.

**Conclusions:**

Addressing gaps in facility readiness and provider competencies for emergency obstetric care, alongside improving coverage of institutional deliveries, is critical to improve maternal outcomes. It is necessary to strengthen providers’ clinical and problem solving skills through capacity building initiatives beyond pre-service training, such as through onsite mentoring and supportive supervision programs. This should be backed by a health systems response to streamline staffing and supply chains in order to improve the quality of emergency obstetric care.

**Electronic supplementary material:**

The online version of this article (doi:10.1186/1471-2393-14-304) contains supplementary material, which is available to authorized users.

## Background

The maternal mortality ratio has declined globally over the past two decades, but it still falls short of the millennium development goal (MDG) target [1]. Only 19 out of 136 developing countries appear to be on track to meet millennium development goal 5 (MDG-5), that targets reduction of the maternal mortality ratio (MMR) by three-quarters between 1990 and 2015 [2]. The global average of maternal mortality is considerably influenced by India alone [2]. India had an MMR of 212/100,000 live births during 2007–09 and it is predicted that this will decrease to 135 by 2015. Thus the proposed MDG target of 109 will not be attained [3, 4]. An analysis of causes of maternal mortality indicates that obstetric haemorrhage, particularly post-partum haemorrhage (PPH), and hypertensive disorders (preeclampsia/eclampsia) are the leading causes of maternal deaths in developing countries [5]. In India, these two conditions contribute to nearly 40% of maternal deaths [6]. Effective interventions exist to address these conditions [7–9]; however, the poor quality of obstetric services provided can contribute to poor outcomes for women and their infants [10].

In the Indian public health system, primary health centres (PHCs) should be available for a rural catchment population of 30,000. “24/7” PHCs are those that offer services around the clock and are staffed by at least one doctor, also known as a medical officer (MO), and 3–5 staff nurses (SNs). These providers offer skilled birth care, including screening, management and referral of maternal and newborn complications to higher-level referral facilities [11, 12]. Referral facilities include public facilities, also termed as first referral units (FRUs) and private facilities; they generally are staffed by a greater number of MOs and SNs. In addition, they usually have specialists, and are equipped with infrastructure and resources to handle obstetric and newborn emergencies [12]. Since 2005, the National Rural Health Mission (NRHM) program has been implemented in India, and has many flexible and innovative strategies to accelerate progress towards reaching MDG-4 and 5[13]. These include: promotion of institutional deliveries through incentive schemes; setting up and strengthening the infrastructure and resources to provide emergency obstetric and newborn care services; mobilization efforts by frontline workers called ASHAs (accredited social health activists)^a^ and strengthening of referral linkages between facilities and communities through ambulance services [13]. As a result of these efforts, there has been a major increase in the number of institutional deliveries over the last few years [14]. However, this has not led to the expected reductions in maternal mortality [15]. Evidence suggests that improved service access and coverage are not enough, and that they need to be complemented by improved service quality for improvements in outcomes [10]. Quality improvement in turn is dependent on the availability of skilled birth attendants, as well as well-functioning health facilities [16].

In this context, we assessed the facility readiness and provider preparedness related to screening and management of the two most common obstetric emergencies, PPH and pre-eclampsia/eclampsia, at both the PHC level and at higher facilities.

## Methods

### Study setting

The present study was conducted in eight districts in northern Karnataka, a southern Indian state that has an overall estimated MMR of 178 per 100,000 live-births [3]. This was done as part of a baseline assessment for a program to provide technical support to the government of Karnataka for improving maternal, neonatal and child health (MNCH) outcomes in this relatively under-developed region. There are large regional disparities in health infrastructure and service delivery between southern and northern Karnataka districts [17]. The eight districts (Bagalkot, Koppal, Bijapur, Bellary, Raichur, Gulbarga, Yadgir and Bidar) of northern Karnataka have a population of 15.1 million (mid-year 2010), comprising about 25% of the state’s population. The female literacy rate is only 42%; the urbanization rate is 25%; and scheduled castes and tribes^b^ comprise 39% of the population in this region. Compared to the rest of the state, this region has a 50% higher crude birth rate (23.4 vs. 15.6 births/ 1000 population) and a 35% higher infant mortality rate (45 vs. 33/1000 live births) [18].

### Study design, study population, sampling and tools

The sampling frame for this cross-sectional study included all primary health centres and higher referral centres (public and private) that offered obstetric care in the eight districts. In this region, there are 403 primary health centres, 111 higher public facilities (including eight government district hospitals, 34 government *taluka or* sub-district hospitals, and 69 community health centres, which are smaller government hospitals), and 193 private health facilities. To achieve a good representation of all types of facilities, 33% of the PHCs, 50% of the community health centres, 100% of *taluka* and district hospitals, and 10 private hospitals per district were randomly selected for the assessment. One facility checklist at each facility and one test case with each cadre of provider in the facility (doctor, staff nurse and specialist/obstetrician) was administered. In facilities with more than one provider, we tried to enroll those that were posted during the day and was free to participate in the study without affecting routine clinical duties. Case sheet audits were also undertaken at the higher facilities. Facility checklists captured infrastructure details about the staff, drugs and equipment related to screening and management of the two complications (see Additional files 1, 2, 3 and 4). Test cases were administered as face-to-face interviews after handing out the case scenarios to the providers (see Additional file 5). Test cases and case sheet audits were designed to capture staff knowledge and practices related to the diagnosis and management of pre-eclampsia/eclampsia and PPH. The data were collected between May and October 2010.

### Facility and provider guidelines for managing preeclampsia/eclampsia and PPH

The study tools were designed to assess if the facilities met standard guidelines for infrastructure and resources; and if the providers followed national protocols related to the management of preeclampsia/eclampsia and PPH [19–21]. The critical resources included availability of functional labour rooms, access to transport services for emergency referrals, drugs such as uterotonics (inj oxytocin, inj methergine or tab misoprostol to enhance contraction of uterus that helps to both prevent and control excess bleeding due to PPH ), anti-hypertensives (tab nifedipine or inj hydralazine to lower blood pressure in pre-eclampsia/eclampsia), anticonvulsants (inj magnesium sulphate or inj diazepam to control the convulsions during an eclamptic attack), intravenous fluids (to manage shock in case of excessive bleeding), laboratory equipment such as a haemoglobinometer (to test the hemoglobin levels), and urine albumin sticks (to detect proteinurea in hypertensive disorders of pregnancy). In addition, first referral units were assessed for facilities to conduct operative deliveries, provision for storing blood, and capability to manage complications that are referred from the PHCs.

Similarly, the national guidelines suggest the following steps of management of the two complications used in the case studies. The initial assessment of a woman presenting with symptoms suggestive of pre-eclampsia/eclampsia includes assessment of blood pressure, testing of urine for proteins, observation for the presence of convulsions and taking the fetal heart rate. A woman with severe pre-eclampsia presents with high B.P (>160/110 mm Hg) and proteinurea; in addition, eclampsia is associated with convulsions, and needs to be managed with oxygen, intravenous fluids, anti-hypertensives and anticonvulsants. Emergency transportation has to be arranged to a facility that can offer follow-up care after initial management of pre-eclampsia/eclampsia, and which has facilities for induction of labour or operative delivery if needed. During follow up care, blood pressure and fluid intake have to be closely monitored, the patient needs to be monitored for convulsions, symptoms related to magnesium toxicity have to be watched for, and the option of inducing labour or operative delivery has to be considered.

PPH is defined as the loss of 500 ml or more of blood during or within 24 hours of birth (referred to as early PPH) and up to six weeks after delivery (referred to as delayed PPH). The most common causes of early PPH include atonic uterus (uterus does not retract after delivery of placenta), retained placenta or placental fragments (placenta not delivered either completely or partially), and genital trauma (vaginal/cervical tears). The initial management of PPH includes assessment and treatment of shock through administration of oxygen and fluids, and specific management of the cause of PPH, followed by referring to a higher facility where blood transfusion and operative delivery, if needed, are available.

### Data collection, approvals and ethics statement

70 field investigators with experience in conducting facility surveys were recruited and trained for 6 days regarding facility guidelines and the use of facility checklists. We also employed 32 field investigators with a medical background for administering the test cases and audits. They received training for a total of 10 days that included both classroom and field practice sessions related to clinical guidelines and the use of various tools. At each facility, the purpose of the study was explained and signed consent obtained from the providers before administering the tools. Ethical approval for the study was obtained from the Health Research Ethics Board, University of Manitoba, Winnipeg, Canada, and the Institutional Ethical Review Board of St. John’s National Academy of Health Sciences, Bangalore, India. Approvals from the government authorities and institution heads were obtained before data collection.

### Data analysis

Data analysis was conducted using STATA, version 12. The extent of facility readiness and provider preparedness related to management of preeclampsia/eclampsia and PPH was the main outcome studied. All three types of higher public facilities were grouped into one category, as they all are expected to function as first referral units for PHCs. The facility audit data were analysed by type of facility to understand the differences in readiness by level of care delivery. The mean availability of providers and proportion of facilities having the required human resources as well as the infrastructure to deal with complications were also examined. The test cases were analysed to observe differences in knowledge regarding the management of complications between the providers at two levels, i.e. by provider cadre and by the facility where providers work. To study differences between providers by cadre, all staff of a particular cadre irrespective of the type of facility were grouped for comparison. Similarly, to assess differences between providers by facility type, all different categories of staff of a particular type of facility were grouped for comparison. Pearson’s chi-square test of significance was applied with 95% confidence intervals.

The current study complies with STROBE guidelines for cross-sectional studies wherever applicable (see Additional file 6).

## Results

### Study coverage

Facility data was available from a total of 131 PHCs, 74 higher public and 74 private facilities, attaining total facility coverage of 96%. Those facilities that were under renovation or those where key informants were not available, as well as a few private hospitals which did not consent to participate, contributed to the gaps in coverage. We were able to administer 475 test cases at these facilities, covering 64.5% of providers. The reasons for non-response were that many staff positions were not sanctioned, or staff were on leave or were deputed elsewhere at the time of data collection. Audits were undertaken in 157 higher facilities, with an attempt to audit five recently managed cases of pre-eclampsia/eclampsia and PPH at each facility. While we therefore expected 785 audits for each complication, only 146 audits of eclampsia and 111 audits of PPH were possible, for a response rate of 18.6% and 14.1% respectively. The poor response rates were largely because of non-availability of case sheets, due to supply chain problems, non-filling of the case sheets in the public facilities, and unwillingness to share case sheets in the private facilities. Because of the poor response rates, results from the case sheet audits are not presented. Table 1 summarizes the sample and coverage details for each of the tools that were administered in the study.

**Table 1:** **Study sample and coverage**

Facility audit								
Facility type		Universe		Sample	Coverage			
PHC		403		133	131		98.5%	
HF-Public		111		77	74		96.1%	
HF-Pvt		193		80	74		92.5%	
Total		707		290	279		96.2%	
**Provider test cases**								
	# Doctors	# Staff nurses	# Specialists	Total # providers	# Facilities	Sample	Coverage	
PHC	1	1	*	2	133	266	204	76.7%
HF-Public	1	1	1	3	77	231	147	63.6%
HF-Pvt	1	1	1	3	80	240	124	51.7%
Total					290	737	475	64.5%
*Specialist positions are not sanctioned at the PHCs								
**Case sheet audit**								
		# Facilities	# Audits planned per facility	Sample	Coverage for pre-eclampsia/eclampsia		Coverage for PPH	
HF-Public		77	5	385	109	28.3%	91	23.6%
HF-Pvt		80	5	400	37	9.3%	20	5.0%
Total		157	5	785	146	18.6%	111	14.1%

HF- Higher Facility; CHC - Community Health Centre; TH – Taluka (sub district) Hospital; DH – District Hospital; PVT - Private Hospital.

### Facility audits

The availability of infrastructure, skilled staff, drugs, equipment and supplies in the management of PPH and pre-eclampsia/eclampsia is shown in Table 2. 84.5% and 62.9% of all facilities had at least one doctor and 3 staff nurses, respectively. The higher public facilities had an average number of 3 doctors and 15 nurses in comparison to private hospitals that had an average number of 2 doctors and 10 staff nurses. Only 33.8% of higher public and 16.2% of private facilities had at least one specialist thus reflecting acute shortage of specialists in the region to deal with emergency care; however these facilities that had an average of 2 specialists per facility. While labour rooms were reported to be highly functional (92%) across all three types of facilities, the private facilities (95%) reported better functionality of operation theatres than public facilities (78%). Only 22% of private facilities had ambulances stationed on their campuses, as compared to 88% of higher public facilities. However, general access, i.e. road connectivity and availability of other means of transport, was high across all the facilities, at about 92%. Uterotonics such as oxytocin and misoprostol were available in less than half of the public facilities at the time of study, while the availability of methergine was better than oxytocin and misoprostol. Magnesium sulphate, the drug of choice to treat eclamptic convulsions, was available in only 18% of PHCs, 48% of higher public facilities and 70% of private facilities. Diazepam, an alternative to magnesium sulphate, was available in 49% of all facilities. Anti-hypertensives such as nifedipine and hydralazine were available in 44% and 11% of all facilities, respectively. The availability of diazepam and nifedipine was higher in private higher facilities (76.3%, 68.3%) compared to public higher facilities (35.6%, 43.8%). Fewer higher public facilities (57%) had Caesarean section equipment when compared to private facilities (84%). Blood storage services were available in 22% of higher public sector and 13% of private facilities, reflecting inadequate capacity for the management of postpartum hemorrhage and related complications in the region.

**Table 2:** **Facility readiness in the public and private health facilities of northern Karnataka**

Facility related parameter	Total (279)	PHC (131)	HF-PUBLIC (74)	HF-PVT (74)
***Human resources***				
Proportion of facilities that had at least one doctor	84.5	80.0	93.2	83.8
Mean no of doctors with Quartiles {Mean(Q1, Q3)} in facilities that had at least one doctor	2(1, 2)	1(1, 1)	3(2, 2)	2(2, 2)
Proportion of facilities that had at three staff nurses	62.9	57.7	75.7	59.5
Mean no of staff nurses with Quartiles {Mean(Q1, Q3)} in facilities that had at least three staff nurses	8(3, 6)	3(3, 3)	15(4, 12)	10(4, 7)
Proportion of facilities that had at least one specialist*	13.3	NA	33.8	16.2
Mean no of specialists with Quartiles {Mean(Q1, Q3)} in facilities that had at least one specialist	2(1, 1)		2(1, 2)	2(1, 1)
***Percent of facilities having the following infrastructure***				
Functional labour rooms	92	85.0	97.2	98.6
Functional operation theatre	60.8	27.2	78.3	95.4
Stationed ambulances	33.0	8.5	87.7	22.4
Access to transportation during emergencies	92.1	96.9	98.6	77.6
***Percent of facilities having the following drugs and supplies***				
Inj oxytocin	60.2	56.2	46.6	80.3
Inj methergine	73.1	66.2	78.1	80.3
Tab misoprostol	47.0	39.2	31.5	75.0
Inj magnesium sulphate	39.8	17.7	47.9	69.7
Tab nifedipine	43.7	29.2	43.8	68.4
Inj hydralazine	11.1	6.9	9.6	19.7
Inj diazepam	48.7	40.0	35.6	76.3
IV fluids (Ringer’s lactate, normal saline, dextrose normal saline)	92.1	98.5	95.9	77.6
Urine albumin sticks	64.2	60.8	80.8	53.9
***Percent of facilities having the following equipment***				
Stethoscope	98.9	98.5	98.6	100.0
Blood pressure machine	98.6	98.5	97.3	100.0
Fetoscope	82.8	77.7	84.9	89.5
Labour table	95.7	96.2	98.6	92.1
Oxygen cylinder with regulator and mask	58.1	33.8	68.5	89.5
Lower caesarean section set	71.1	NA	57.5	84.2
Blood bag refrigerators	17.4	NA	21.9	13.2
Hemoglobinometer	80.6	73.1	91.8	82.9

HF- Higher Facility; CHC - Community Health Centre; TH – Taluka (sub district) Hospital; DH – District Hospital; PVT - Private Hospital.

*The specialist positions exist only in the higher public and private facilities.

### Provider preparedness

The findings related to provider knowledge about screening and management of pre-eclampsia/eclampsia are summarized in Table 3. The majority of the providers did not identify a need for assessing level of consciousness nor for testing urine for protein during initial assessment (considered essential for diagnosis and management); 78.5% (95% CI, 74.6-82.1) of the providers diagnosed severe pre-eclampsia correctly; 72.6% (95% CI, 68.4-76.6) would administer anti-hypertensives; and only 46% (95% CI, 41.8-50.9) would administer magnesium sulphate for the management of severe pre-eclampsia. For eclampsia management, only 24% (95% CI, 20.4-28.3) would administer oxygen; 54.1% (95% CI, 49.5-58.7) and 65.1% (95% C.I, 60.6-69.3) of providers would administer anti-hypertensives and magnesium sulphate respectively; 14% (95% C.I, 11.1-17.6) would record inputs–outputs; and 18% (95% C.I, 14.7-21.9) would monitor for the toxicity of magnesium sulphate as a part of follow-up care for eclampsia. In comparison with higher facilities, PHCs performed poorly across many knowledge parameters, but significant differences were observed in the administration of magnesium sulphate for severe preeclampsia and administration of oxygen (p = 0.001), and use of anti-hypertensives for eclampsia (p < 0.05). Compared to doctors and specialists, staff nurses had poorer knowledge related to management of pre-eclampsia/eclampsia and most of the differences were statistically highly significant (p < 0.001).

**Table 3:** **Test case findings about knowledge of providers in diagnosing and managing pre-eclampsia/eclampsia by facility and provider type**

Knowledge parameter assessed through the test case	Total (475)	CI [95% Conf. Interval]	PHC (204)	HF-PUBLIC (147)	HF-PVT (124)	P Value*	Nurse/ANM (248)	Doctors (138)	Obstetricians (89)	P Value**
***Initial assessment for pre-eclampsia/eclampsia***										
Check blood pressure	94.9	92.6-96.7	96.1	92.5	96.0	0.269	92.7	97.8	96.6	0.066
Assess consciousness	6.1	4.1-8.7	3.4	8.2	8.1	0.107	3.6	6.5	12.4	0.012
Measure fetal heart rate	66.7	62.3-71	67.2	63.3	70.2	0.480	56.9	72.5	85.4	<0.001
Assess urine for protein	55.2	50.6-59.7	51.0	57.1	59.7	0.260	38.3	65.9	85.4	<0.001
***Diagnosis and management of severe pre-eclampsia***										
Diagnose severe pre-eclampsia	78.5	74.6-82.1	78.4	83.0	73.4	0.159	67.7	85.5	97.8	<0.001
Administer magnesium sulphate	46.3	41.8-50.9	36.8	51.7	55.6	0.001	30.2	50.7	84.3	<0.001
Administer anti-hypertensive drugs if diastolic BP > 110 mm Hg	72.6	68.4-76.6	69.6	71.4	79.0	0.165	67.7	74.6	83.1	0.016
Immediately refer to higher facility	34.5	30.3-39	51.0	33.3	8.9	<0.001	39.9	42.8	6.7	<0.001
***Diagnosis and management of eclampsia***										
Diagnose eclampsia	80.4	76.6-83.9	77.9	86.4	77.4	0.089	67.7	92.0	97.8	<0.001
Administer oxygen	24.2	20.4-28.3	16.2	33.3	26.6	0.001	14.9	29.0	42.7	<0.001
Administer magnesium sulphate	65.1	60.6-69.3	61.3	66	70.2	0.251	51.2	69.6	96.6	<0.001
Administer anti-hypertensive drugs if diastolic BP > 110 mm Hg	54.1	49.5-58.7	47.1	58.5	60.5	0.027	44.8	58.0	74.2	<0.001
***Follow up after initial management of eclampsia***										
Administer repeat dose of magnesium sulphate if woman is still seizing	32.6	28.4-37.1	27.9	36.1	36.3	0.167	19.4	36.2	64.0	<0.001
Administer anti-hypertensive drugs if diastolic BP > 110 mm Hg	51.4	46.8-55.9	49.5	51.7	54.0	0.726	45.2	54.3	64.0	0.007
Induce labour	30.5	26.4-34.9	20.1	40.1	36.3	<0.001	21.8	25.4	62.9	<0.001
Maintain intake/output record	14.1	11.1-17.6	11.8	17.0	14.5	0.375	8.9	15.2	27.0	<0.001
Monitor magnesium sulphate toxicity	18.1	14.7-21.9	16.7	18.4	20.2	0.724	12.9	18.1	32.6	<0.001

HF- Higher Facility; CHC - Community Health Centre; TH – Taluka (sub district) Hospital; DH – District Hospital; PVT - Private Hospital; ANM – Auxiliary Nurse Midwife.

*Pearson’s chi-square test for independence between providers by facilities.

**Pearson’s chi-square test for independence between providers by provider type.

The findings related to provider knowledge about screening and management of PPH are summarized in the Table 4. Only 37.7% (95% CI, 33.3-42.2) of the providers would check uterine tone during initial assessment; 39% (95% CI, 34.3-43.3) mentioned that uterine atony is the most common cause of early PPH; 40% (95% CI, 35.8-44.8) correctly defined early PPH; 36% (95% CI, 31.7-40.5) would perform a speculum examination for PPH with a contracted uterus; and 18% (95% CI, 14.4-21.4) would recommend a hemoglobin test as a part of the overall management of the complication. Providers at higher facilities performed better than those in the PHCs, and statistically significant differences were observed in knowledge related to uterine atony (p < 0.001), performing speculum examination in cases of PPH with contracted uterus (<0.001), knowledge related to hemoglobin investigations (<0.05), and monitoring vital signs (<0.01). Specialists performed much better than other staff. Staff nurses, who are the major care providers in these settings, were very deficient in knowledge regarding the diagnosis and management of PPH.

**Table 4:** **Test case findings about knowledge of providers in diagnosing and managing postpartum hemorrhage by facility and provider type**

Knowledge parameter assessed through the test case	Total	CI [95% Conf. Interval]	PHC (204)	HF-PUBLIC (147)	HF-PVT (124)	P Value*	Nurse/ ANM (248)	Doctors (138)	Obstetricians (89)	P Value**
Identify first action - checking uterine tone	37.7	33.3-42.2	27.9	44.2	46.0	0.001	25.4	33.3	78.7	<0.001
List uterine atony as a common cause of PPH	38.7	34.3-43.3	27.9	44.2	50.0	<0.001	18.5	50.7	76.4	<0.001
List retained placenta as a common cause of PPH	54.9	50.3-59.5	52.9	56.5	56.5	0.748	46.0	63.0	67.4	<0.001
List vaginal or cervical tears as a common cause of PPH	60.0	55.4-64.4	59.8	63.3	56.5	0.520	54.4	60.1	75.3	0.003
Correctly define early PPH	40.2	35.8-44.8	43.6	38.1	37.1	0.414	30.6	45.7	58.4	<0.001
Correctly diagnose genital trauma as a cause of PPH	53.3	48.7-57.8	45.6	53.7	65.3	0.002	36.7	59.4	89.9	<0.001
Perform a speculum examination for PPH with contracted uterus	36.0	31.7-40.5	25.5	44.2	43.5	<0.001	26.2	30.4	71.9	<0.001
Check vital signs as a part of the initial assessment	68.2	63.8-72.4	65.7	70.7	69.4	0.574	62.1	71.7	79.8	0.005
Prescribe starting IV	55.2	50.6-59.7	54.4	56.5	54.8	0.927	49.6	54.3	71.9	0.001
List blood hemoglobin as an essential test required	17.7	14.4-21.4	11.8	21.1	23.4	0.012	9.3	17.4	41.6	0.002
Monitor vital signs	59.2	54.6-63.6	52.0	59.2	71.0	0.003	51.6	59.4	79.8	<0.001

HF- Higher Facility; CHC - Community Health Centre; TH – Taluka (sub district) Hospital; DH – District Hospital; PVT - Private Hospital; ANM – Auxiliary Nurse Midwife; PPH – Postpartum hemorrhage; IV – Intravenous.

*Pearson’s chi-square test for independence between providers by facilities.

**Pearson’s chi-square test for independence between providers by provider type.

## Discussion

In this study, conducted in an area of India with generally poor MNCH outcomes, we attempted to assess the quality of emergency obstetric care for PPH and pre-eclampsia/eclampsia at primary and first referral unit level. Prior to this study, no assessments of this nature and scale had been attempted, and hence we had no sense about the status or quality of emergency obstetric care in the region. We covered a large sample of facilities and providers, and had reasonably good coverage of the private sector. This is probably the first study in the region that has assessed the status of emergency obstetric care in private facilities. We assessed both facility readiness and provider preparedness. The facility audits revealed gaps in the availability of human resources, and in the availability of certain key equipment, drugs and supplies. As a study in Uganda concluded, the number and availability of health care providers in the labour room affects quality of care [22]. Shortages in staff and supplies can significantly affect the quality of maternal and newborn care, and the World Health Organization (WHO) recommends that investments be made both in new skills development as well as in facilities, equipment and infrastructure [16, 23]. Although the NRHM in India has contributed to the strengthening of infrastructure and resources in the public sector at large, there are deficiencies in human resources, drugs and supplies that are vital to deal with the two most common obstetric emergencies, PPH and pre-eclampsia/eclampsia. The problem is exacerbated when there is inequitable distribution of emergency obstetric care facilities between the district and sub-districts [18]. A WHO study that assessed emergency obstetric care across six developing countries, including India, indicated that public facilities were unable to provide emergency obstetric care due to lack of good management systems to ensure continuous availability of drugs and supplies [16]. While the lack of availability of specialist physicians was consistent across facilities, private facilities seemed to have better availability of emergency care-related equipment and drugs. It may be that the reported private facility staff data in the study was an underestimate. Typically, in India, the private sector is very large and unregulated, and approximately 80% of trained physicians work in the private sector [24].

The competency assessments revealed knowledge gaps in relation to the diagnosis and management of PPH and pre-eclampsia. While health workers at PHCs had the poorest knowledge scores, health workers at the higher-level public and private facilities, which are the referral centres for PHCs, also had significant knowledge gaps. Of the three types of providers, the staff nurses had the most severe knowledge gaps. This is concerning, as they are generally the first point of contact for emergency care in both first level and higher facilities. Poor competency in staff nurses can contribute to delays in receiving adequate care after reaching facilities [25]. Poor availability of emergency obstetric care in public facilities has been found to be due to a lack of competency and skills among providers [16]. We also observed that private providers did not perform much better than public providers in the management of the two obstetric emergencies, and similar observations have been made elsewhere in India [26]. This raises concerns about the effectiveness of public-private partnerships, which have been advocated in recent years: the NRHM has provisions to contract private specialists to provide emergency care in public facilities, but unless their competencies can be addressed through capacity building programs or continuing medical education, these efforts may not have much effect in improving the quality of emergency care. Most public sector staff nurses and doctors have received induction training pertaining to maternal care, but this does not seem to have adequately addressed gaps in knowledge and practice. This suggests that one-off training sessions have not been very effective in sustaining providers’ knowledge and good practices, and continuous education and mentoring may therefore be needed [27].

Our study has some limitations. In relation to assessing provider competencies, direct observation of skills and practice might have given a more accurate picture than just assessing the knowledge domain. However, this was not feasible in the rural Indian context, where individual facilities, particularly PHCs, do not experience a high volume of cases. Furthermore, case sheet audits can reflect providers’ current practice and adherence to management standards, and thus offer more opportunities to understand quality of care, particularly in low resource settings such as Tanzania and Thailand [28, 29]. However, we experienced poor coverage of audits due to non-availability of case sheets and poor documentation practices within practice settings. The existing case sheets, when further investigated, were not found to be useful as job-aids, nor were audit friendly, as they were open-ended, not uniformly introduced in all facilities at the time of data collection, and left to the discretion of the provider to document. Hence, the case sheets were very poorly completed, resulting in missed opportunities for us to understand the practice of the providers. These deficiencies in case sheets and their use themselves warrant attention from program managers, to ensure standardized and evidence-based practices in health facilities, that can be assessed through case sheet audits.

## Conclusions

There is a clear need to improve critical infrastructure and resources, as well as provider competencies related to emergency obstetric care, particularly PPH and pre-eclampsia/eclampsia, to complement efforts to increase the coverage of institutional deliveries. A comprehensive skills-building program for providers should be implemented, to address not just clinical competencies, but also skills related to management and problem solving in the context of the limited infrastructure and resources that providers have to work with. There is also a need to provide case sheets that are user-friendly, can serve as job-aids and can be easily audited. Some of these issues could be addressed through strategies such as supportive supervision and enhanced clinical mentoring, as follow-up after one-time pre-service training [27]. Such strategies can be effective in small-scale projects [30–32], but whether they can work at large scale needs to be evaluated. There is also a need to explore newer and non-threatening ways of measuring quality of care within the private sector; this assumes particular importance in the context of increasing public-private partnerships in maternal care. A multi-pronged approach is therefore needed to improve the quality of emergency obstetric care, and to accelerate India’s progress toward millennium development goals.

## Endnotes

^a^Accredited Social Health Activists (ASHAs) are field level workers who act in a health promotion capacity, creating awareness and counseling women on safe delivery, and facilitating access to health care services.

^b^Scheduled caste and tribes are among the most disadvantaged socio-economic groups in India.

## Author’s information

Krishnamurthy Jayanna: Director, Sukshema (MNCH) project, Karnataka Health Promotion Trust, Bangalore, India. Krishnamurthy@khpt.org

^1^ Project Sukshema, Karnataka Heath Promotion Trust, Bangalore, India

^2^ Department of Community Health Sciences, Faculty of Medicine, University of Manitoba, Winnipeg, Canada

^3^ Division of Epidemiology & Population Health, St John’s Research Institute, St John’s National Academy of Health Sciences

^4^ Department of Obstetrics, St John’s Medical College & Hospital, St John’s National Academy of Health Sciences

## Electronic supplementary material

Additional file 1: **FACILITY AUDIT_PHC, PDF (file name and format).** (DOC 3 MB)

Additional file 2: **FACILITY AUDIT_CHC, PDF.** (DOC 3 MB)

Additional file 3: **FACILITY AUDIT_PVT, PDF.** (DOC 370 KB)

Additional file 4: **FACILITY AUDIT_TH_DH, PDF.** (DOC 358 KB)

Additional file 5: **TEST CASE STUDY_PE_PPH, PDF.** (DOCX 32 KB)

Additional file 6: **Completed STROBE Checklist.** (DOC 86 KB)

## Abbreviations

PPH: Postpartum hemorrhage
MDG: Millennium development goal
MMR: Maternal mortality ratio
PHC: Primary health centre
FRU: First referral unit
NRHM: National rural health mission
ASHA: Accredited social health activist
MNCH: Maternal newborn and child health
HF: Health facility
CHC: Community health centre
TH: Taluka hospital
DH: District hospital
PVT: Private
C.I: Confidence interval
WHO: World Health Organization.
