# Supplementary material for: Assessment of facility readiness and provider preparedness for dealing with postpartum haemorrhage and pre-eclampsia/eclampsia in public and private health facilities of northern Karnataka, India: a cross-sectional study
Source: BMC Pregnancy Childbirth. 2014 Sep 4;14:304. doi: 10.1186/1471-2393-14-304 (PMC4161844; doi:10.1186/1471-2393-14-304)
Supplement: Supplementary file 1 — Additional file 1: FACILITY AUDIT_PHC, PDF (file name and format). (DOC 3 MB) [file 12884_2014_1171_MOESM1_ESM.doc]

**MAPPING MNCH SERVICES**

**PRIMARY HEALTH CENTRE (PHC)** FORMAT

| A: IDENTIFICATION | | | | | | | |
| --- | --- | --- | --- | --- | --- | --- | --- |
| DISTRICT ____________________________________________________________________________________________________  TALUK_______________________________________________________________________________________________________  PRIMARY HEALTH CENTRE(Location)________________________________________________________________________  DISTRICT HOSPITAL/TALUK HOSPITAL/CHC (Location)__________________________________________________________  NUMBER OF SCs CATERED BY THE PHC .......................................................................................................................................................  WHETHER DESIGNATED PRIMARY HEALTH UNIT (YES=1, NO=2)…………………………………………………….…….…..  WHETHER DESIGNATED 24x7 PHC? (YES=1, NO=2)……………………………………………………………………….…….…..  NUMBER OF VISITS MADE……………………………………………………………………………………………………….…….…..  NAME OF THE INTERVIEWER______________________________________________________________________  SIGNATURE OF THE INTERVIEWER________________________________________________________________  NAME OF THE PHC MO _________________________________________________________________________  SIGNATURE OF THE PHC MO _____________________________________________________________________ | | | | | | | |
|  | VISIT 1 | | | VISIT 2 | | VISIT 3 | |
| DATE | DAY MONTH YEAR | | | DAY MONTH YEAR | | DAY MONTH YEAR | |
| RESULT* |  | | |  | |  | |
| RESULT: 1. Completed 2. Primary respondent not available 3. Postponed 4. Refused 5. Partly completed 6. Other_____________  (SPECIFY) | | | | | | | |
|  | | SPOT CHECKED BY | FIELD EDITED BY | | OFFICE EDITED BY | | KEYED BY |
| NAME | |  |  | |  | |  |
| CODE | |  |  | |  | |  |
| DATE | |  |  | |  | |  |

Namaskar! My name is ______________, working with the Karnataka Health Promotion Trust, Bangalore in the MNCH project which aims to help the Government of Karnataka to achieve the NRHM goals of improving maternal, neonatal and child health outcomes. Mapping of the various maternal, neonatal and child health services are undertaken in 8 districts specifically to identify gaps in service availability and accessibility. I request you to kindly help me in completing this mapping form for your PHC. The information required includes the details of the SCs covered by your PHC, physical infrastructure, equipments, drugs and supplies, specific MNCH services available, selected service statistics related to your PHC, and about the receipt and utilization of untied funds. It will take about 30 minutes to complete this format. I request you to provide the most honest and correct information, as this will help your district to prepare an evidence-based project implementation plan to secure better services for the rural poor.

**B: HUMAN RESOURCES**

**1. How many staff positions are sanctioned in your PHC (including Group D staff)?**

| **SN** | **2.** Please give me the designation of each staff sanctioned. **RECORD THE DESIGNATIONS OF EACH SACTIONED POSITION IN SEPARATE ROWS. FOR EXAMPLE, IF 3 MEDICAL OFFICERS POSITIONS ARE SANCTIONED, RECORD MEDICAL OFFICER 1 IN ONE ROW, MEDICAL OFFICER 2 IN THE ANOTHER ROW, MEDICAL OFFICER 3 IN YET ANOTHER ROW.** | 3. Is this position currently available at the PHC?  (AVAILABLE = 1,  NOT AVAILABLE = 2)  **ASK Q.4-8 IF AVAILABLE. IF NOT AVAILABLE, ASK Q.9-10** | 4. Name of the staff | 5. Is the available staff a male or a female?  (MALE = 1,  FEMALE = 2) | 6. What is his / her educational  qualification?  **RECORD AS REPORTED** | 7. What is the type of appointment of the staff in this position?  (PERMANENT /REGULAR APPOINTMENT AT CURRENT HEALTH FACILITY=1  ON DEPUTATION FROM ANOTHER GOVERNMENT FACILITY=2  SHARED WITH OTHER GOVERNMENT FACILITIES=3  CONTRACTED FULL TIME=4  CONTRACTED PART TIME=5  OTHER (SPECIFY)=6) | 8. Does the [STAFF] resides in PHC headquarter? (YES IN PHC STAFF QUARTER=1, YES IN OWN/RENTED HOUSE IN PHC HQ=2, NO=3) | 9. Why is this position currently not available at the PHC?  (NOT RECRUITED /APPOINTED=1  ON DEPUTATION TO ANOTHER HEALTH FACILITY=2  ON LEAVE / PURSUING HIGHER EDUCATION OR TRAINING MORE THAN 6 MONTHS=3  NOT REPORTING FOR DUTY =4  OTHER (SPECIFY)=5) | 10. How long has this position been vacant?  **RECORD DURATION IN MONTHS, RECORD 96 IF MORE THAN 96 MONTHS** |
| --- | --- | --- | --- | --- | --- | --- | --- | --- | --- |
| **1** |  |  |  |  |  |  |  |  |  |
| **2** |  |  |  |  |  |  |  |  |  |
| **3** |  |  |  |  |  |  |  |  |  |
| **4** |  |  |  |  |  |  |  |  |  |
| **5** |  |  |  |  |  |  |  |  |  |
| **6** |  |  |  |  |  |  |  |  |  |
| **SN** | **Q2** | **Q3** | **Q4** | **Q5** | **Q6** | **Q7** | **Q8** | **Q9** | **Q10** |
| **7** |  |  |  |  |  |  |  |  |  |
| **8** |  |  |  |  |  |  |  |  |  |
| **9** |  |  |  |  |  |  |  |  |  |
| **10** |  |  |  |  |  |  |  |  |  |
| **11** |  |  |  |  |  |  |  |  |  |
| **12** |  |  |  |  |  |  |  |  |  |
| **13** |  |  |  |  |  |  |  |  |  |
| **14** |  |  |  |  |  |  |  |  |  |
| **15** |  |  |  |  |  |  |  |  |  |
| **16** |  |  |  |  |  |  |  |  |  |
| **17** |  |  |  |  |  |  |  |  |  |
| **18** |  |  |  |  |  |  |  |  |  |

**C: PHYSICAL INFRASTRUCTURE**

| Q # | Question | **RECORD 1 IF YES AND 2 IF NO** |
| --- | --- | --- |
| 11 | Is the PHC building owned by the government? |  |
| 12 | Is there a separate labour room in the PHC?  **RECORD BY OBSERVATION** | **IF NO, SKIP TO Q19** |
| 13 | Is the labour room functional 24x7? |  |
| 14 | How many tables are there in the labour room?  RECORD BY OBSERVATION | **RECORD #** |
| 15 | Does the labour room have piped water supply 24×7?  RECORD BY OBSERVATION |  |
| 16 | Is there soap for hand wash in labour room?  RECORD BY OBSERVATION |  |
| 17 | Does the labour room have a functioning electricity powered lamp?  RECORD BY OBSERVATION |  |
| 18 | Does the labour room have generator/inverter back-up?  RECORD BY OBSERVATION |  |
| 19 | Does the labour room have neonatal corner?  RECORD BY OBSERVATION |  |
| 20 | Does the PHC have Operation Theatre (OT)?  **RECORD BY OBSERVATION** | **IF NO, SKIP TO Q23** |
| 21 | Is the OT functional 24x7? |  |
| 22 | Have any operations been performed in the OT in last 3 months? |  |
| 23 | Are Caesarian sections conducted in the OT? |  |
| 24 | Is there a separate laboratory in the PHC?  **RECORD BY OBSERVATION** |  |
| 25 | Is there a pharmacy for drug storage and dispensing in the PHC?  **RECORD BY OBSERVATION** |  |
| 26 | Is there a functional telephone connection at the PHC? |  |
| 27 | Does the PHC have a computer? |  |
| 28 | Does the PHC have access to internet? |  |
| 29 | Does the PHC have an ambulance? |  |
| 30 | Does the PHC have access to vehicle ( 108 or others) for transporting patients during emergencies? |  |

**D: EQUIPMENTS**

| SL # | General equipments | | 31A. Are the following equipments available in the labour room of the PHC and are they currently functional? **RECORD AVAILABILITY BY OBSERVATION AND FUNCTIONALITY AS REPORTED**  (AVAILABLE AND FUNCTIONAL=1, AVAILABLE BUT NOT FUNCTIONAL=2, NOT AVAILABLE=3) |
| --- | --- | --- | --- |
| 1  2  3  4 | Stethoscope …………………………………………………………………………….  Blood Pressure machine ….…………………………………………………………………  Fetoscope ……………………………………………………………………………..  Adult weighing scale ………………………………………………………………………… | | |
| SL # | Labour room equipments | | 31B. Are the following equipments available in the labour room of the PHC and are they currently functional? **RECORD AVAILABILITY BY OBSERVATION AND FUNCTIONALITY AS REPORTED**  (AVAILABLE AND FUNCTIONAL=1, AVAILABLE BUT NOT FUNCTIONAL=2, NOT AVAILABLE=3) |
| 5  6  7  8  9  10  11  12  13  14  15 | Labour Table……………………………………………………………………………..  Lamp/ light ……………………..…………………………………………………………  Oxygen Cylinder with regulator and Mask…………………………………………….  Foot-operated/ electrical suction………………………………………………………  Emergency drug and equipment tray/ trolley………………………………………..  Normal Delivery Kit……………………………………………………………………..  Vacuum cup and suction apparatus………………………………………………….  Obstetric Forceps……………………………………………………………………….  MVA syringe and cannula ………………………..…………………………………..  High pressure sterilizer / Autoclave ………………………………………………………  Suture Kit ………………………………………………………………………………… | | |
| Newborn child care Unit equipments | | | 31C. Are the following equipments available in newborn child care unit of the PHC and are they currently functional? **RECORD AVAILABILITY BY OBSERVATION AND FUNCTIONALITY AS REPORTED**  (AVAILABLE AND FUNCTIONAL=1, AVAILABLE BUT NOT FUNCTIONAL=2, NOT AVAILABLE=3) |
| 16  17  18  19  20  21 | Self inflating bag (Ambu bag) and mask neonatal size ..…………………………………  Endotracheal intubation tubes (neonatal)….……………………………………………  Laryngoscope (neonatal) ……………………………………………………………….  Feeding tubes for baby….. ………………………………………………………………..  Radiant warmer/incubator ………………………………………………………………..  Infant weighing scale……………………………………………………………………… | | |
| Cold chain equipments | | 31D. Are the following cold chain related equipments available in the PHC and are they currently functional? **RECORD AVAILABILITY BY OBSERVATION AND FUNCTIONALITY AS REPORTED**  (AVAILABLE AND FUNCTIONAL=1, AVAILABLE BUT NOT FUNCTIONAL=2, NOT AVAILABLE=3) | |
| 22  23  24 | Ice Lined Refrigerator (Large/Small)…………………………………………………..  Deep Freezer (Large/Small) / Refrigerator………………………………………………  Vaccine carrier…………………………………………………………………………….. | | |
| Laboratory equipments | | 31E. Are the following laboratory equipments available in the PHC and are they currently functional? **RECORD AVAILABILITY BY OBSERVATION AND FUNCTIONALITY AS REPORTED**  (AVAILABLE AND FUNCTIONAL=1, AVAILABLE BUT NOT FUNCTIONAL=2, NOT AVAILABLE=3) | |
| 25  26 | Hemoglobinometer…………………………………………………………………………..  Binocular / Monocular microscope…………………………………………………………… | | |

**E: DRUGS & SUPPLIES**

| Sl # | Drugs/supplies | 32. Is [DRUG/SUPPLY) currently available at the PHC? **OBSERVE AND RECORD** (CURRENTLY AVAILABLE=1, GENERALLY AVAILABLE, BUT CURRENTLY NOT=2, WAS NEVER AVAILABLE=3)  **ASK Q32 FOR ITEMS WITH CODES 1 OR 2** | 33. Was there any stock out of [DRUG/SUPPLY] for more than 15 days in the past 2 months? (YES=1, NO=2) |
| --- | --- | --- | --- |
| 1  2  3  4  5  6  7  8  9  10  11  12  13  14  15  16  17  18  19  20  21  22  23  24  25  26  27  28  29  30  31  32  33  34  35  36  37  38  39  40  41  42  43  44  45  46  47 | T. Iron & Folic acid (Large) (FST/FAT) ……………………………………………….. …  Inj. Oxytocin (Syntocinon / Pitocin) …………………………………………………………  Inj. Methergine/ Methyl ergometrine ………………………………….…………………..  T Misoprostol / Inj. Prostodin ………………………………..………………………………  Inj. Magnesium Sulphate ……………………………………………………………………  Inj. Betamethasone / Dexamethasone… …………………………………………………  T. Nifedipine / Alpha dopa………………………………………………………………….  Inj. Hydralazine ……………………………………………………………………………..  Inj Furosemide (Lasix) ……………………………………………………………………..  Inj Diazepam.....................................................................................................................  Inj. Phenobarbitone ………………………………………..………………………………  S Cotrimoxazole/ Amoxycillin……………………………………………………………  C. Amoxycillin ……………………………………………………………………………….  Inj. Ampicillin……………………………………………………………………………………  Inj. Penicilin…………………………………………………………………………………….  Inj. Gentamycin ..............................................................................................................  Inj. Metronidazole … ……………………………………………………………………………  T. Chloroquine 150 mg ………………………………………………………………………  T. Albendazole /Mebendazole ………………………………………………………………  T Nevirapine ……………………………………………………………………………………  S Nevirapine..............................................................................................................  T Paracetamol / Ibuprofen / Diclofenac (Voveran)…………………………………………  Inj Paracetamol / Diclofenac Sodium (Voveran).................................................................  Inj. Adrenaline……………………………………………………………………………………  Inj. Xylocaine / Lidocaine / Lignocaine ……………………………….. ……………………  ORS packets…………………………………………………………………………………  Ringer Lactate / NS / DNS (500 ml.)………………………………………………………  10% or 25% Dextrose ampoules…………………………..……………………..…………  Inj. TT vaccine ………………………………………………………………………………  Inj. BCG Vaccine ………………………………………………………………………………  Oral Polio Vaccine (OPV)...........................................................................................  Inj. DPT Vaccine …………………………………………………………………………..  Inj. Measles Vaccine …………………………………………………………………………  Condoms ……………………………………………………………………………………….  Oral Contraceptive pills (OCP, Mala D,  Mala N, Centchroman).......................................................................................................  Injectable contraceptives……………………………………………………………………  IUCD (Copper T)………………………………………………………………………………..  Urethral catheters ……………………………………………………………………………  IV cannulas ………………………………………………………………………………..  Disposable/AD syringes ……………………………………………………………………  Disposable Gloves ……………………………………………………………………………….  Urine albumin/sugar strips …………………………………………………………………….  Urine pregnancy test kits ………………………………………………………………………  ABO & Rh antibodies reagents………………………………………………………………  HIV Test Kits……………………………………………………………………………………  ANC cards (Thaayi cards)………………………………………………………………….  Under-5/Immunization cards ……………………………………………………………….. | | |

**F: SERVICES**

| Sl # | ADVICE/COUNSELING SERVICES DURING PREGNANCY | 34A. Please provide a list of advice/ counseling services provided to pregnant women during antenatal period. **FOR EACH SERVICE SPONTANEOUSLY MENTIONED, RECORD 1. FOR ITEMS NOT MENTIONED SPONTANEOUSLY, ASK Q35A, LEAVING THE CELL BLANK.** **FOR SERVICES SPONTANEOUSLY MENTIONED, SKIP TO Q36A.** | 35A. Is [SERVICE] provided to the pregnant women during antenatal period? (Yes=1, No=2) **ONLY** **FOR ITEMS CODED 1 IN THIS COLUMN, ASK Q36A.** | 36A. Generally who provides the [SERVICE]? **PUT √ AGAINST ALL PERSONS PROVIDING THE SERVICE** | | | | | | |
| --- | --- | --- | --- | --- | --- | --- | --- | --- | --- | --- |
|  |  |  |  | Doctor | Nurse | | | Others (Specify) | | |
| 1 | Registration |  |  |  |  | | |  | | |
| 2 | ANC checkups |  |  |  |  | | |  | | |
| 3 | Promotion of delivery by skilled personnel |  |  |  |  | | |  | | |
| 4 | Danger signs of pregnancy & when to seek care |  |  |  |  | | |  | | |
| 5 | Breastfeeding Counseling |  |  |  |  | | |  | | |
| 6 | HIV Counseling |  |  |  |  | | |  | | |
| 7 | Nutrition advice |  |  |  |  | | |  | | |
| 8 | Contraceptive Counseling |  |  |  |  | | |  | | |
| ANTENATAL SERVICES | | 34B. Please provide a list of antenatal services provided to pregnant women. **FOR EACH SERVICE SPONTANEOUSLY MENTIONED, RECORD 1. FOR ITEMS NOT MENTIONED SPONTANEOUSLY, ASK Q35B, LEAVING THE CELL BLANK. FOR SERVICES SPONTANEOUSLY MENTIONED, SKIP TO Q36B.** | 35B. Is [SERVICE] provided to the pregnant women?  (Yes=1, No=2) **ONLY** **FOR ITEMS CODED 1 IN THIS COLUMN, ASK Q36B.** | 36B. Generally who provides the [SERVICE]? **PUT √ AGAINST ALL PERSONS PROVIDING THE SERVICE** | | | | | | |
|  |  |  |  | Doctor | | Nurse | | | Others (Specify) | |
| 9 | Identification of high risk pregnancies |  |  |  | |  | | |  | |
| 10 | Identification of danger signs of pregnancy |  |  |  | |  | | |  | |
| 11 | Weight measurement |  |  |  | |  | | |  | |
| 12 | Blood pressure measurement |  |  |  | |  | | |  | |
| 13 | Abdominal examination |  |  |  | |  | | |  | |
| 14 | Listening to Fetal heart sounds |  |  |  | |  | | |  | |
| 15 | Urine dipstick for protein |  |  |  | |  | | |  | |
| 16 | Urine dipstick/ microscopy for UTI |  |  |  | |  | | |  | |
| 17 | Hemoglobin estimation |  |  |  | |  | | |  | |
| 18 | Blood grouping and Rh typing |  |  |  | |  | | |  | |
| 19 | Oral Glucose Tolerance testing (OGTT) for diabetes |  |  |  | |  | | |  | |
| 20 | Syphilis testing |  |  |  | |  | | |  | |
| 21 | HIV testing |  |  |  | |  | | |  | |
| 22 | Iron/folate supplementation |  |  |  | |  | | |  | |
| 23 | Tetanus toxoid (TT) immunization |  |  |  | |  | | |  | |
| 24 | Provision of de-worming tablets (Albendazole /Mebendazole) |  |  |  | |  | | |  | |
| 25 | Malaria prophylaxis with T.Chloroquine |  |  |  | |  | | |  | |
| 26 | PMTCT provision (Nevirapine for HIV positive mothers and babies) |  |  |  | |  | | |  | |
| 27 | Referral services |  |  |  | |  | | |  | |
| INTRAPARTUM CARE | | 34C. Please provide a list of services provided during labour and delivery. **FOR EACH SERVICE SPONTANEOUSLY MENTIONED, RECORD 1. FOR ITEMS NOT MENTIONED SPONTANEOUSLY, ASK Q35C, LEAVING THE CELL BLANK. FOR SERVICES SPONTANEOUSLY MENTIONED, SKIP TO Q36C.** | 35C. Is [SERVICE] provided during labour and delivery?  (Yes=1, No=2) **ONLY** **FOR ITEMS CODED 1 IN THIS COLUMN, ASK Q36C.** | 36C. Generally who provides the [SERVICE]? **PUT √ AGAINST ALL PERSONS PROVIDING THE SERVICE** | | | | | | |
|  |  |  |  | Doctor | | | Nurse | | | Others (Specify) |
| 28 | Clean delivery |  |  |  | | |  | | |  |
| 29 | Use of Partograph |  |  |  | | |  | | |  |
| 30 | IV access |  |  |  | | |  | | |  |
| 31 | Oxytocin for labour induction or augmentation |  |  |  | | |  | | |  |
| 32 | Identification of danger signs during labour |  |  |  | | |  | | |  |
| 33 | Assisted delivery (Vacuum/Forceps) |  |  |  | | |  | | |  |
| 34 | Oxygen |  |  |  | | |  | | |  |
| 35 | IM/IV Antibiotics |  |  |  | | |  | | |  |
| 36 | IV Furosemide |  |  |  | | |  | | |  |
| 37 | Inj Magnesium sulfate |  |  |  | | |  | | |  |
| 38 | Manual removal of placenta |  |  |  | | |  | | |  |
| 39 | Caesarean Section |  |  |  | | |  | | |  |
| 40 | Blood transfusion |  |  |  | | |  | | |  |
| 41 | Referral services |  |  |  | | |  | | |  |

| POST-PARTUM (WITHIN 24 HOURS) | | 34D. Please provide a list of services provided immediately after delivery. **FOR EACH SERVICE SPONTANEOUSLY MENTIONED, RECORD 1. FOR ITEMS NOT MENTIONED SPONTANEOUSLY, ASK Q35D, LEAVING THE CELL BLANK**. **FOR SERVICES SPONTANEOUSLY MENTIONED, SKIP TO Q36D.** | 35D. Is [SERVICE] provided immediately after delivery?  (Yes=1, No=2) **ONLY** **FOR ITEMS CODED 1 IN THIS COLUMN, ASK Q36D.** | 36D. Generally who provides the [SERVICE]? **PUT √ AGAINST ALL PERSONS PROVIDING THE SERVICE** | | |
| --- | --- | --- | --- | --- | --- | --- |
|  |  |  |  | Doctor | Nurse | Others (Specify) |
| 42 | Blood pressure measurement |  |  |  |  |  |
| 43 | Routine use of uterotonics (Methergine, Oxytocin, Misoprostol) |  |  |  |  |  |
| 44 | Estimate amount of blood loss |  |  |  |  |  |
| 45 | Uterine massage for severe bleeding |  |  |  |  |  |
| 46 | Initiation of immediate Breastfeeding |  |  |  |  |  |
| 47 | Identification & management of early postpartum complications |  |  |  |  |  |
| 48 | Referral services |  |  |  |  |  |
| POST-NATAL SERVICES | | 34E. Please provide a list of post natal services. **FOR EACH SERVICE SPONTANEOUSLY MENTIONED, RECORD 1. FOR ITEMS NOT MENTIONED SPONTANEOUSLY, ASK Q35E, LEAVING THE CELL BLANK. FOR SERVICES SPONTANEOUSLY MENTIONED, SKIP TO Q36E.** | 35E. Is [SERVICE] provided to a recent delivery?  (Yes=1, No=2) **ONLY** **FOR ITEMS CODED 1 IN THIS COLUMN, ASK Q36E.** | 36E. Generally who provides the [SERVICE]? **PUT √ AGAINST ALL PERSONS PROVIDING THE SERVICE** | | |
|  |  |  |  | Doctor | Nurse | Others (Specify) |
| 49 | Lochia (Vaginal discharge after delivery) examination |  |  |  |  |  |
| 50 | Identification and management of danger signs of late postpartum complications |  |  |  |  |  |
| 51 | Contraceptive/family planning counseling |  |  |  |  |  |
| 52 | Counseling on danger signs in neonate & when to seek care |  |  |  |  |  |
| 53 | Counseling on infant immunization |  |  |  |  |  |
| 54 | Counseling on late postpartum complication & when to seek care |  |  |  |  |  |
| 55 | Provision of contraceptives |  |  |  |  |  |
| 56 | Referral Services |  |  |  |  |  |

| POST-ABORTION SERVICES | | 34F. Please provide a list of post-abortion services provided. **FOR EACH SERVICE SPONTANEOUSLY MENTIONED, RECORD 1. FOR ITEMS NOT MENTIONED SPONTANEOUSLY, ASK Q35F, LEAVING THE CELL BLANK. FOR SERVICES SPONTANEOUSLY MENTIONED, SKIP TO Q36F.** | 35F. Is [SERVICE] related to abortion provided?  (Yes=1, No=2) **ONLY** **FOR ITEMS CODED 1 IN THIS COLUMN, ASK Q36F.** | 36F. Generally who provides the [SERVICE]? **PUT √ AGAINST ALL PERSONS PROVIDING THE SERVICE** | | |
| --- | --- | --- | --- | --- | --- | --- |
|  |  |  |  | Doctor | Nurse | Others (Specify) |
| 56 | D & C (Dilatation & Curettage) |  |  |  |  |  |
| 57 | MVA (Manual vacuum aspiration) |  |  |  |  |  |
| 58 | Provision of Antibiotics |  |  |  |  |  |
| 59 | Identification of danger signs/complications |  |  |  |  |  |
| 60 | Contraceptive/family planning counseling |  |  |  |  |  |
| 61 | Provision of contraceptives |  |  |  |  |  |
| 62 | Referral services |  |  |  |  |  |
| NEO-NATAL CARE SERVICES | | 34G. Please provide a list of neonatal care services provided. **FOR EACH SERVICE SPONTANEOUSLY MENTIONED, RECORD 1. FOR ITEMS NOT MENTIONED SPONTANEOUSLY, ASK Q35G, LEAVING THE CELL BLANK. FOR SERVICES SPONTANEOUSLY MENTIONED, SKIP TO Q36G.** | 35G. Is [SERVICE] provided to newborns?  (Yes=1, No=2) **ONLY** **FOR ITEMS CODED 1 IN THIS COLUMN, ASK Q36G.** | 36G. Generally who provides the [SERVICE]? **PUT √ AGAINST ALL PERSONS PROVIDING THE SERVICE** | | |
|  |  |  |  | Doctor | Nurse | Others (Specify) |
| 63 | Clean cord care |  |  |  |  |  |
| 64 | Drying & Warmth / Kangaroo Mother Care |  |  |  |  |  |
| 65 | Oral & Nasal Suction |  |  |  |  |  |
| 66 | Bag & Mask ventilation |  |  |  |  |  |
| 67 | Chest Compressions (CPR) |  |  |  |  |  |
| 68 | Weigh baby |  |  |  |  |  |
| 69 | Measure length of baby |  |  |  |  |  |
| 70 | Oral Polio/BCG Vaccine before discharge |  |  |  |  |  |
| 71 | Identification of neonatal danger signs/illness |  |  |  |  |  |
| 72 | Oxygen |  |  |  |  |  |
| 73 | Nasogastric feeds |  |  |  |  |  |
| 74 | IV fluids |  |  |  |  |  |
| 75 | Emollient therapy |  |  |  |  |  |
| 76 | IM/IV Antibiotics |  |  |  |  |  |
| 77 | Stabilization of ill infant (Use of IV fluids, anti seizure medication) |  |  |  |  |  |
| 78 | Referral Services |  |  |  |  |  |
| 79 | Neonatal check-up on day 2 |  |  |  |  |  |
| 80 | Neonatal check-up on day 4 |  |  |  |  |  |
| CHILD HEALTH SERVICES | | 34H. Please provide a list of child health services provided. **FOR EACH SERVICE SPONTANEOUSLY MENTIONED, RECORD 1. FOR ITEMS NOT MENTIONED SPONTANEOUSLY, ASK Q35H, LEAVING THE CELL BLANK. FOR SERVICES SPONTANEOUSLY MENTIONED, SKIP TO Q36H.** | 35H. Is [SERVICE] provided to children?  (Yes=1, No=2) **ONLY** **FOR ITEMS CODED 1 IN THIS COLUMN, ASK Q36H.** | 36H. Generally who provides the [SERVICE]? **PUT √ AGAINST ALL PERSONS PROVIDING THE SERVICE** | | |
|  |  |  |  | Doctor | Nurse | Others (Specify) |
| 81 | Use of growth chart for weight recording |  |  |  |  |  |
| 82 | Child Immunization |  |  |  |  |  |
| 83 | Screening for pneumonia |  |  |  |  |  |
| 84 | Antibiotics for ARI |  |  |  |  |  |
| 85 | Assessment of dehydration |  |  |  |  |  |
| 86 | Measures weight |  |  |  |  |  |
| 87 | Measures height |  |  |  |  |  |
| 88 | Referral Services |  |  |  |  |  |

**G: SERVICE STATISTICS**

| **Q #** | **Question** | **Jan, 2010** | **Feb, 2010** | **Mar, 2010** | **April 2009 to March 2010** | **Base document for the indicator** |
| --- | --- | --- | --- | --- | --- | --- |
| 37 | # of pregnant women registered for ANC at PHC |  |  |  |  |  |
| 38 | # of deliveries conducted at PHC |  |  |  |  |  |
| 39 | # of pregnant women referred to higher centres for care |  |  |  |  |  |
| 40 | # of live-births at PHC |  |  |  |  |  |
| 41 | # of infants who have received Measles vaccine at PHC |  |  |  |  |  |
| 42 | # of neo-natal deaths (≤28 days) at PHC |  |  |  |  |  |
| 43 | # of post-neonatal (>=29 days and <12 months) deaths at PHC |  |  |  |  |  |
| 44 | # of maternal deaths at PHC |  |  |  |  |  |
| 45 | # of beneficiaries of JSY (Janani Suraksha Yojana) scheme at PHC |  |  |  |  |  |
| 46 | # of beneficiaries of Madilu scheme at PHC |  |  |  |  |  |
| 47 | # of beneficiaries of Prasuti Araike scheme in the PHC |  |  |  |  |  |
| 48 | # of beneficiaries of Yashasvini scheme in the PHC |  |  |  |  |  |
| 49 | Was any maternal death audit carried out from your PHC in year 2009-2010? | | | | YES………………………1  NO………………………..2 | |
| 50 | Was any infant death audit carried out from your PHC in year 2009-2010? | | | | YES………………………1  NO………………………..2 | |
| 51 | Is a Rogi Kalyana Samiti / Arogya Raksha Samiti formed at the PHC? | | | | YES………………………1  Q53  NO………………………..2 | |
| 52 | When was the Rogi Kalyana Samiti / Arogya Raksha Samiti formed at the PHC? | | | | DAY  MONTH  YEAR | |

**H: UNTIED FUNDS**

| Q# | Question | Coding categories |
| --- | --- | --- |
| 53 | Has the PHC received the untied funds for the year 2009-2010? | YES………………………….1  END  NO…………………………...2 |
| 54 | What was the amount received as untied fund for the year 2009-2010? | |  |  |  |  |  |  | | --- | --- | --- | --- | --- | --- | |
| 55 | When was this amount received? | DAY  MONTH  YEAR |
| 56 | So far, how much of the untied fund received for 2009-10 has been utilized? |  |
| 57 | What are the items for which the fund was utilized? Please give the list of items and the amount utilized for each item. AMOUNT  ITEM  1_______________________________________________________  2_______________________________________________________  3_______________________________________________________  4_______________________________________________________  5_______________________________________________________ | |

**THANKS FOR GIVING YOUR PRECIOUS TIME**
