# Supplementary material for: Assessment of facility readiness and provider preparedness for dealing with postpartum haemorrhage and pre-eclampsia/eclampsia in public and private health facilities of northern Karnataka, India: a cross-sectional study
Source: BMC Pregnancy Childbirth. 2014 Sep 4;14:304. doi: 10.1186/1471-2393-14-304 (PMC4161844; doi:10.1186/1471-2393-14-304)
Supplement: Supplementary file 4 — Additional file 4: FACILITY AUDIT_TH_DH, PDF. (DOC 358 KB) [file 12884_2014_1171_MOESM4_ESM.doc]

**MAPPING MNCH SERVICES**

**PRIVATE HOSPITAL/NURSING HOME FORMAT**

| A: IDENTIFICATION DISTRICT ____________________________________________________________________________________________________  TALUK_______________________________________________________________________________________________________  TOWN/CITY NAME_________________________________________________________________________________  NUMBER OF VISITS MADE……………………………………………………………………………………………………….…….…..  NAME OF THE INTERVIEWER______________________________________________________________________  SIGNATURE OF THE INTERVIEWER________________________________________________________________  NAME OF THE HOSPITAL MEDICAL SUPERINTENDENT ______________________________________________________  SIGNATURE OF THE HOSPITAL MEDICAL SUPERINTENDENT _________________________________________________ | | | | | | | |
| --- | --- | --- | --- | --- | --- | --- | --- |
|  | VISIT 1 | | | VISIT 2 | | VISIT 3 | |
| DATE | DAY MONTH YEAR | | | DAY MONTH YEAR | | DAY MONTH YEAR | |
| RESULT* |  | | |  | |  | |
| RESULT: 1. Completed 2. Primary respondent not available 3. Postponed 4. Refused 5. Partly completed 6. Other_____________  (SPECIFY) | | | | | | | |
|  | | SPOT CHECKED BY | FIELD EDITED BY | | OFFICE EDITED BY | | KEYED BY |
| NAME | |  |  | |  | |  |
| CODE | |  |  | |  | |  |
| DATE | |  |  | |  | |  |
|  | | | | | | | |

Namaskara! My name is ______________, working with the Karnataka Health Promotion Trust, Bangalore in the MNCH project which aims to help the Government of Karnataka to achieve the NRHM goals of improving maternal, neonatal and child health outcomes. Mapping of the various maternal, neonatal and child health services is undertaken in 8 districts specifically to identify gaps in service availability and accessibility. I request you to kindly help me in completing this mapping form for your HOSPITAL. The information required includes the details of the human resources, physical infrastructure, equipments, drugs and supplies, specific MNCH services available, selected service statistics related to your HOSPITAL, and about the receipt and utilization of untied funds. It will take about 30 minutes to complete this format. I request you to provide the most honest and correct information, as this will help your district to prepare an evidence-based project implementation plan to secure better services for the rural poor.

**B: HUMAN RESOURCES**

| Sl # | 1. Please give me a list of the designations of different staff that are currently in positiion at your Hospital/ Nursing Home. RECORD THE DESIGNATIONS FIRST. | | 2. How many staff in [CADRE] are currently in position / visiting consultants at your HOSPITAL? RECORD #. |
| --- | --- | --- | --- |
|
|
| 1 |  |  | |
| 2 |  |  | |
| 3 |  |  | |
| 4 |  |  | |
| 5 |  |  | |
| 6 |  |  | |
| 7 |  |  | |
| 9 |  |  | |
| 10 |  |  | |
| 13 |  |  | |
| 14 |  |  | |
| 15 |  |  | |
| 16 |  |  | |
| 17 |  |  | |
| 18 |  |  | |
| 19 |  |  | |

**C: PHYSICAL INFRASTRUCTURE**

| Q# | Question | **RECORD 1 IF YES AND 2 IF NO** |
| --- | --- | --- |
| 3 | Is the hospital building owned by the government? |  |
| 4 | Is a separate unit for Obstetrics & Gynecology present in the OPD of hospital? **RECORD BY OBSERVATION** |  |
| 5 | Is a separate unit for Pediatrics Gynecology present in the OPD of hospital? **RECORD BY OBSERVATION** |  |
| 6 | Is there a separate labour room in the hospital?  **RECORD BY OBSERVATION** | **IF NO, SKIP TO Q14** |
| 7 | Is the labour room functional 24x7? |  |
| 8 | How many tables are there in the labour room?  RECORD BY OBSERVATION | **RECORD #** |
| 9 | Does the labour room have piped water supply 24×7?  RECORD BY OBSERVATION |  |
| 10 | Is there soap for hand wash in labour room?  RECORD BY OBSERVATION |  |
| 11 | Does the labour room have a functioning electricity powered lamp?  RECORD BY OBSERVATION |  |
| 12 | Does the labour room have generator/inverter back-up?  RECORD BY OBSERVATION |  |
| 13 | Does the labour room have neonatal corner?  RECORD BY OBSERVATION |  |
| 14 | Does the HOSPITAL have Operation Theatre?  **RECORD BY OBSERVATION** | **IF NO, SKIP TO Q23** |
| 15 | Is the OT functional 24x7? |  |
| 16 | Has any operation been performed in the OT in the last 3 months? |  |
| 17 | Are Caesarian sections conducted in the OT? |  |
| 18 | How many tables are there in OT?  RECORD BY OBSERVATION | **RECORD #** |
| 19 | Does the OT have piped water supply 24×7?  RECORD BY OBSERVATION |  |
| 20 | Is there soap for hand wash in OT?  RECORD BY OBSERVATION |  |
| 21 | Does the OT have a functioning electricity powered lamp?  RECORD BY OBSERVATION |  |
| 22 | Does the OT have generator/inverter back-up?  RECORD BY OBSERVATION |  |
| 23 | Is there a separate laboratory in the hospital?  **RECORD BY OBSERVATION** |  |
| 24 | Is there a blood storage unit in the hospital?  **RECORD BY OBSERVATION** | **IF NO, SKIP TO Q26** |
| 25 | Is the blood storage facility functional 24x7? |  |
| 26 | Is there a pharmacy for drug storage and dispensing in the HOSPITAL? **RECORD BY OBSERVATION** |  |
| 27 | Is an AYUSH wing attached to the hospital?  **RECORD BY OBSERVATION** |  |
| 28 | Is there a separate emergency/casualty room in the hospital?  **RECORD BY OBSERVATION** |  |
|  | Question | **RECORD 1 IF YES AND 2 IF NO** |
| 29 | Is there a separate sick newborn care unit/Nursery in the hospital?  **RECORD BY OBSERVATION** | **IF NO, SKIP TO Q31** |
| 30 | How many beds are there in the sick newborn care unit/Nursery?  **RECORD BY OBSERVATION** | **RECORD #** |
| 31 | Does the hospital have separate wards for men and women?  **RECORD BY OBSERVATION** |  |
| 32 | In total, how many beds are available at the hospital?  **RECORD BY OBSERVATION** | **RECORD #** |
| 33 | How many beds are there for men?  **RECORD BY OBSERVATION** | **RECORD #** |
| 34 | How many beds are there for women?  **RECORD BY OBSERVATION** | **RECORD #** |
| 35 | How many antenatal beds are available?  **RECORD BY OBSERVATION** | **RECORD #** |
| 36 | How many postnatal beds are available?  **RECORD BY OBSERVATION** | **RECORD #** |
| 37 | How many pediatric beds are available at the hospital?  **RECORD BY OBSERVATION** | **RECORD #** |
| 38 | Is there a functional telephone connection at the hospital? |  |
| 39 | Does the hospital have a computer? |  |
| 40 | Does the hospital have access to internet? |  |
| 41 | Does the hospital have an ambulance (other than 108)? |  |
| 42 | Does the hospital have access to vehicle during emergencies? |  |

**D: EQUIPMENTS**

| SL # | General equipments | | 43A. Are the following equipments available in the labour room of the PHC and are they currently functional? **RECORD AVAILABILITY BY OBSERVATION AND FUNCTIONALITY AS REPORTED**  (AVAILABLE AND FUNCTIONAL=1, AVAILABLE BUT NOT FUNCTIONAL=2, NOT AVAILABLE=3) |
| --- | --- | --- | --- |
| 1  2  3  4 | Stethoscope …………………………………………………………………………….  Blood Pressure machine ….…………………………………………………………………  Fetoscope ………………………………………………………………………………  Adult weighing scale ………………………………………………………………………… | | |
| SL # | Labour room equipments | | 43B. Are the following equipments available in the labour room of the hospital and are they currently functional? **RECORD AVAILABILITY BY OBSERVATION AND FUNCTIONALITY AS REPORTED**  (AVAILABLE AND FUNCTIONAL=1, AVAILABLE BUT NOT FUNCTIONAL=2, NOT AVAILABLE=3) |
| 5  6  7  8  9  10  11  12  13  14 | Labour Table……………………………………………………………………………..  Lamp / light ……………………………………………………………………………….  Oxygen Cylinder with regulator and Mask…………………………………………….  Foot-operated/ electrical suction………………………………………………………  Emergency drug and equipment tray/ trolley………………………………………..  Normal Delivery Kit……………………………………………………………………..  Vacuum cup and suction apparatus………………………………………………….  Obstetric Forceps……………………………………………………………………….  High pressure sterilizer / Autoclave…………………………………………………..  Suture Kit………………………………………………………………………………… | | |
| Newborn child care Unit equipments | | | 43C. Are the following equipments available in newborn child care unit of the hospital and are they currently functional? **RECORD AVAILABILITY BY OBSERVATION AND FUNCTIONALITY AS REPORTED**  (AVAILABLE AND FUNCTIONAL=1, AVAILABLE BUT NOT FUNCTIONAL=2, NOT AVAILABLE=3) |
| 15  16  17  18  19  20  21  22  23  24  25  26  27 | Mucus extractor with foot-operated / electrical suction……………………………………  Self inflating bag (Ambu bag) and mask-neonatal size…………………………………  Oxygen hood (neonatal) ………………………………………………………………..  Laryngoscope (neonatal)……………………………………………………………….  Endotracheal intubation tubes (neonatal)……………………………………………..  Laryngeal mask airway (2 ½, 3, 4 sizes)……………………………………………..  Meconium aspirator……………………………………………………………………..  Pulse oximeter……………………………………………………………………………  Infant Feeding tubes (Nasogastric tubes (8, 10, 12 FG))……………………………  Phototherapy unit ………………………………………………………………………  Radiant warmer/incubator ……………………………………………………………  Nebuliser/ MDI (Metered dose inhaler ………………………………………………  Infant weighing scale …………………………………………………………….…… | | |
| Operation Theatre Equipments | | 43D. Are the following equipments available in the OT of the hospital and are they currently functional? **RECORD AVAILABILITY BY OBSERVATION AND FUNCTIONALITY AS REPORTED**  (AVAILABLE AND FUNCTIONAL=1, AVAILABLE BUT NOT FUNCTIONAL=2, NOT AVAILABLE=3) | |
| 28  29  30  31  32  33  34  35 | Operation table……………………………………………………………………………  Foot-operated or electrical suction apparatus…………………………………………..  Oxygen cylinder with regulator and mask……………………………………………..  Cardiac monitor………………………………………………………………………….  Pulse oximeter …………………………………………………………………………..  Diathermy equipment ……………………………………………………………………  Emergency drug and equipment tray/trolley …………………………………….……  High pressure sterilizer / Autoclave…………………………………………………… | | |
| Anesthetic equipments | | 43E. Are the following anesthetist equipments available in the hospital and are they currently functional? **RECORD AVAILABILITY BY OBSERVATION AND FUNCTIONALITY AS REPORTED**  (AVAILABLE AND FUNCTIONAL=1, AVAILABLE BUT NOT FUNCTIONAL=2, NOT AVAILABLE=3) | |
| 36  37  38  39  40  41  42  43  44 | Oropharyngeal Airway (adult)………………………………………………………………….  Endotracheal tubes (adult size)………………………………………………………………  Laryngoscope set with adult blades ……………………………………………………..  Face masks…………………………………………………………………………………  Spinal needle SS 4 2 set…………………………………………………………………..  Anesthesia machine ………………………………………………………………………  Boyles Apparatus ………………………………………………………………………….  Nitrous oxide cylinder ………………………………………………………………………  Halothane / Isoflurane /Enflurane vaporizer ………………………………………………… | | |
| Surgical equipments | | 43F. Are the following surgical equipments available in the hospital and are they currently functional? **RECORD AVAILABILITY BY OBSERVATION AND FUNCTIONALITY AS REPORTED**  (AVAILABLE AND FUNCTIONAL=1, AVAILABLE BUT NOT FUNCTIONAL=2, NOT AVAILABLE=3) | |
| 45  46  47  48 | Sterilization Set……………………………………………………………………………..  D & C Set…………………………………………………………………………………….  LSCS set……………………………………………………………………………………..  MVA syringe and cannula……………………………………………………………….. | | |
| Cold chain equipments | | 43G. Are the following cold chain related equipments available in the hospital and are they currently functional? **RECORD AVAILABILITY BY OBSERVATION AND FUNCTIONALITY AS REPORTED**  (AVAILABLE AND FUNCTIONAL=1, AVAILABLE BUT NOT FUNCTIONAL=2, NOT AVAILABLE=3) | |
| 49  50 | Ice Lined Refrigerator (Large/Small)…………………………………………………..  Deep Freezer (Large/Small) ……………………………………………… | | |
| Blood storage and transfusion equipments | | 43H. Are the following blood storage and transfusion equipments available in the hospital and are they currently functional? **RECORD AVAILABILITY BY OBSERVATION AND FUNCTIONALITY AS REPORTED**  (AVAILABLE AND FUNCTIONAL=1, AVAILABLE BUT NOT FUNCTIONAL=2, NOT AVAILABLE=3) | |
| 51  52 | Blood bag refrigerators…………………………………………………………………..  Insulated carrier boxes with ice packs…………………………………………………. | | |
| Radiology equipments | | 43I. Are the following radiology equipments available in the HOSPITAL and are they currently functional? **RECORD AVAILABILITY BY OBSERVATION AND FUNCTIONALITY AS REPORTED**  (AVAILABLE AND FUNCTIONAL=1, AVAILABLE BUT NOT FUNCTIONAL=2, NOT AVAILABLE=3) | |
| 53  54 | Ultra sonogram…………………………………………………………………………  X ray unit………………………………………………………………………………… | | |

| Laboratory equipments | | 43J. Are the following laboratory equipments available in the HOSPITAL and are they currently functional? **RECORD AVAILABILITY BY OBSERVATION AND FUNCTIONALITY AS REPORTED**  (AVAILABLE AND FUNCTIONAL=1, AVAILABLE BUT NOT FUNCTIONAL=2, NOT AVAILABLE=3) |
| --- | --- | --- |
| 55  56  57  58  59 | Hemoglobinometer………………………………………………………………………..  Binocular microscope…………………………………………………………………….  Electrolytes testing equipment……………………………………………………………  Glucometer/Dextrosticks…………………………………………………………………..  Bilirubinometer……………………………………………………………………………… | |

**E: DRUGS & SUPPLIES**

| Sl # | Drugs/supplies | 44. Is [DRUG/SUPPLY) currently available at the HOSPITAL? **OBSERVE AND RECORD** (CURRENTLY AVAILABLE=1, GENERALLY AVAILABLE, BUT CURRENTLY NOT=2, WAS NEVER AVAILABLE=3)  **ASK Q45 FOR ITEMS WITH CODES 1 OR 2** | 45. Was there any stock out of [DRUG/SUPPLY] for more than 15 days in the past 2 months? (YES=1, NO=2) |
| --- | --- | --- | --- |
| 1  2  3  4  5  6  7  8  9  10  11  12  13  14  15  16  17  18  19  20  21  22  23  24  25  26  27  28  29  30  31  32  33  34  35  36  37  38  39  40  41  42  43  44  45  46  47  48  49  50  51  52  53  54  55  56 | T. Iron & Folic acid (IFA) Large (FST/FAT)  Inj Iron dextran / Iron sorbitol……………………………………………………………..  Inj. Oxytocin (Syntocinon / Pitocin) ………………………………………………..………  Inj. Methergine/ Methyl ergometrine  T Misoprostol / Inj. Prostodin………………………………………………………………  Inj. Magnesium Sulphate…………………………………..……………………………..  Inj. Betamethasone / Dexamethasone …………………………………..…………………  T. Nifedipine / Alpha dopa …………………………………………………………………  Inj. Hydralazine ………………………………………………………………………………..  Inj Furosemide (Lasix) ………………………………………………………………………..  Inj Diazepam………………………………………………………………………………..  Inj. Phenobarbitone…………………………………………………………………………..  Inj Pethidine (Meperidine)……………………………………………………………………  S Cotrimoxazole/ Amoxycillin… … ………………………………………………………  C. Amoxycillin …………………………………………………………………………………..  Inj. Ampicillin………………………………………………………………………………….  Inj. Penicilin ……………………………………………………………………………………  Inj. Gentamycin ………………………………………………………………………………..  Inj. Metronidazole … ……………………………………………………………………………  T. Chloroquine 150 mg ………………………………………………………………………  T. Albendazole /Mebendazole ……………………………………………………………..  T Nevirapine ……………………………………………………………………………………  S Nevirapine.....................................................................................................................  T Paracetamol / Ibuprofen / Diclofenac (Voveran)………………………………………  Inj Paracetamol / Diclofenac Sodium (Voveran)…………………………………………..  Inj. Adrenaline…………………………………………………………………………………..  Inj. Dopamine………………………………………………………………………………….  Inj. Xylocaine (Lidocaine / Lignocaine)………………………………………………………  Inj. Bupivacaine …………………………………………………………………………………  Inj. Thiopentone (Pentothal) / Ketamine / Propofol…………………………………………  Isoflurane / Enflurane / Halothane ……………………………………………………………  ORS packets………………………………………………..………………………………  Ringer Lactate / NS / DNS (500 mL) ……………………………………………………….  10% or 25% Dextrose ampoules……………………………………………………………  Colloids (HAES – Starch 6,  Venofundin Hemaccel)………………………………………………………………………  Isolyte P (Pediatric IV fluids) ……………………………………………………………………  Inj. TT vaccine …………………………………………………………………………………..  Inj. BCG Vaccine ………………………………………………………………………………..  Oral Polio Vaccine (OPV) ..........................................................................................  Inj. DPT Vaccine …………………………………………………………………………..  Inj. Measles Vaccine …………………………………………………………………………  Condoms ………………………………………………………………………………………  Oral Contraceptive pills (OCP, Mala D,  Mala N, Centchroman)………………………………………………………………………….  Injectable contraceptives……………………………………………………………………  IUCD (Copper T)………………………………………………………………………………  Urethral catheters …………………………………………………………………………  IV cannulas for children (22G/24G)………………………………………………………  IV cannulas for adults (16G/18G)………………………………………………………………  Disposable/AD syringes ……………………………………………………………………  Disposable Gloves …………………………………………………………………………  Urine albumin/sugar strips …………………………………………………………………….  Urine pregnancy test kits ………………………………………………………………..  ABO & Rh antibodies reagents……………………………………………………………  HIV Test Kits…………………………………………………………………………………..  ANC cards (Thaayi cards)………………………………………………………………………  Under-5/Immunization cards ………………………………………………………………..TT | | |

**G: SERVICES**

| Sl # | | ADVICE/COUNSELING SERVICES DURING PREGNANCY | 46A. Please provide a list of advice/ counseling services provided to pregnant women during antenatal period. **FOR EACH SERVICE SPONTANEOUSLY MENTIONED, RECORD 1. FOR ITEMS NOT MENTIONED SPONTANEOUSLY, ASK Q47A, LEAVING THE CELL BLANK.** **FOR SERVICES SPONTANEOUSLY MENTIONED, SKIP TO Q48A.** | 47A. Is [SERVICE] provided to the pregnant women during antenatal period? (Yes=1, No=2) **ONLY** **FOR ITEMS CODED 1 IN THIS COLUMN, ASK Q48A.** | 48A. Generally who provides the [SERVICE]? **PUT √ AGAINST ALL PERSONS PROVIDING THE SERVICE** | | | | | | | | | |  |
| --- | --- | --- | --- | --- | --- | --- | --- | --- | --- | --- | --- | --- | --- | --- | --- |
|  |  |  |  |  | Doctor | Nurse | | | | | Others (Specify) | | | |  |
| 1 | | Registration |  |  |  |  | | | | |  | | | |  |
| 2 | | ANC checkups |  |  |  |  | | | | |  | | | |  |
| 3 | | Promotion of delivery by skilled personnel |  |  |  |  | | | | |  | | | |  |
| 4 | | Danger signs of pregnancy |  |  |  |  | | | | |  | | | |  |
| 5 | | Breastfeeding Counseling |  |  |  |  | | | | |  | | | |  |
| 6 | | HIV Counseling |  |  |  |  | | | | |  | | | |  |
| 7 | | Nutrition advice |  |  |  |  | | | | |  | | | |  |
| 8 | | Contraceptive Counseling |  |  |  |  | | | | |  | | | |  |
| ANTENATAL SERVICES | | | 46B. Please provide a list of antenatal services provided to pregnant women. **FOR EACH SERVICE SPONTANEOUSLY MENTIONED, RECORD 1. FOR ITEMS NOT MENTIONED SPONTANEOUSLY, ASK Q47B, LEAVING THE CELL BLANK. FOR SERVICES SPONTANEOUSLY MENTIONED, SKIP TO Q48B.** | 47B. Is [SERVICE] provided to the pregnant women?  (Yes=1, No=2) **ONLY** **FOR ITEMS CODED 1 IN THIS COLUMN, ASK Q48B.** | 48B. Generally who provides the [SERVICE]? **PUT √ AGAINST ALL PERSONS PROVIDING THE SERVICE** | | | | | | | | | |  |
|  |  |  |  |  | Doctor | | | Nurse | | | | | Others (Specify) | |  |
| 9 | | Identification of high risk pregnancies |  |  |  | | |  | | | | |  | |  |
| 10 | | Identification of danger signs of pregnancy |  |  |  | | |  | | | | |  | |  |
| 11 | | Weight measurement |  |  |  | | |  | | | | |  | |  |
| 12 | | Blood pressure Measurement |  |  |  | | |  | | | | |  | |  |
| 13 | | Abdominal examination |  |  |  | | |  | | | | |  | |  |
| 14 | | Listening to Fetal heart sounds |  |  |  | | |  | | | | |  | |  |
| 15 | | Urine dipstick for protein |  |  |  | | |  | | | | |  | |  |
| 16 | | Urine dipstick/ microscopy for UTI |  |  |  | | |  | | | | |  | |  |
| 17 | | Hemoglobin estimation |  |  |  | | |  | | | | |  | |  |
| 18 | | Blood grouping and Rh typing |  |  |  | | |  | | | | |  | |  |
| 19 | | Oral Glucose Tolerance testing (OGTT) for diabetes |  |  |  | | |  | | | | |  | |  |
| 20 | | Syphilis testing |  |  |  | | |  | | | | |  | |  |
| 21 | | HIV testing |  |  |  | | |  | | | | |  | |  |
| 22 | | Iron/folate supplementation |  |  |  | | |  | | | | |  | |  |
| 23 | | Tetanus toxoid immunization |  |  |  | | |  | | | | |  | |  |
| 24 | | Provision of de-worming tablets (Albendazole /Mebendazole) |  |  |  | | |  | | | | |  | |  |
| 25 | | Malaria prophylaxis with T.Chloroquine |  |  |  | | |  | | | | |  | |  |
| 26 | | PMTCT provision (Nevirapine for HIV positive mothers and babies) |  |  |  | | |  | | | | |  | |  |
| 27 | | Referral services |  |  |  | | |  | | | | |  | |  |
| INTRAPARTUM CARE | | | 46C. Please provide a list of services provided during labour and delivery. **FOR EACH SERVICE SPONTANEOUSLY MENTIONED, RECORD 1. FOR ITEMS NOT MENTIONED SPONTANEOUSLY, ASK Q47C, LEAVING THE CELL BLANK. FOR SERVICES SPONTANEOUSLY MENTIONED, SKIP TO Q48C.** | 47C. Is [SERVICE] provided during labour and delivery?  (Yes=1, No=2) **ONLY** **FOR ITEMS CODED 1 IN THIS COLUMN, ASK Q48C.** | 48C. Generally who provides the [SERVICE]? **PUT √ AGAINST ALL PERSONS PROVIDING THE SERVICE** | | | | | | | | | | |
|  |  |  |  |  | Doctor | | | | Nurse | | | | | Others (Specify) | |
| 28 | | Clean delivery |  |  |  | | | |  | | | | |  | |
| 29 | | Use of Partograph |  |  |  | | | |  | | | | |  | |
| 30 | | IV access |  |  |  | | | |  | | | | |  | |
| 31 | | Oxytocin for labour induction or augmentation |  |  |  | | | |  | | | | |  | |
| 32 | | Identification of danger signs during labour |  |  |  | | | |  | | | | |  | |
| 33 | | Assisted delivery (Vacuum/Forceps) |  |  |  | | | |  | | | | |  | |
| 34 | | Oxygen |  |  |  | | | |  | | | | |  | |
| 35 | | IM/IV Antibiotics |  |  |  | | | |  | | | | |  | |
| 36 | | IV Furosemide |  |  |  | | | |  | | | | |  | |
| 37 | | Inj Magnesium sulfate |  |  |  | | | |  | | | | |  | |
| 38 | | Tocolytics for pre-term labour |  |  |  | | | |  | | | | |  | |
| 39 | | Steroids for pre-term labour |  |  |  | | | |  | | | | |  | |
| 40 | | Manual removal of placenta |  |  |  | | | |  | | | | |  | |
| 41 | | Blood transfusion |  |  |  | | | |  | | | | |  | |
| 42 | | Caesarean Section |  |  |  | | | |  | | | | |  | |
| 43 | | Referral services |  |  |  | | | |  | | | | |  | |
| POST-PARTUM (WITHIN 24 HOURS) | | | 46D. Please provide a list of services provided immediately after delivery. **FOR EACH SERVICE SPONTANEOUSLY MENTIONED, RECORD 1. FOR ITEMS NOT MENTIONED SPONTANEOUSLY, ASK Q47D, LEAVING THE CELL BLANK**. **FOR SERVICES SPONTANEOUSLY MENTIONED, SKIP TO Q48D.** | 47D. Is [SERVICE] provided immediately after delivery?  (Yes=1, No=2) **ONLY** **FOR ITEMS CODED 1 IN THIS COLUMN, ASK Q48D.** | 48D. Generally who provides the [SERVICE]? **PUT √ AGAINST ALL PERSONS PROVIDING THE SERVICE** | | | | | | | | | | |
|  |  |  |  |  | Doctor | | | | Nurse | | | | | Others (Specify) | |
| 44 | | Blood pressure measurement |  |  |  | | | |  | | | | |  | |
| 45 | | Routine use of uterotonics (Methergine, Oxytocin, Misoprostol) |  |  |  | | | |  | | | | |  | |
| 46 | | Estimate amount of blood loss |  |  |  | | | |  | | | | |  | |
| 47 | | Uterine massage for severe bleeding |  |  |  | | | |  | | | | |  | |
| 48 | | Initiation of immediate Breastfeeding |  |  |  | | | |  | | | | |  | |
| 49 | | Identification & management of early postpartum complications |  |  |  | | | |  | | | | |  | |
| 50 | | Referral services |  |  |  | | | |  | | | | |  | |
| POST NATAL SERVICES | | | 46E. Please provide a list of post natal services. **FOR EACH SERVICE SPONTANEOUSLY MENTIONED, RECORD 1. FOR ITEMS NOT MENTIONED SPONTANEOUSLY, ASK Q47E, LEAVING THE CELL BLANK. FOR SERVICES SPONTANEOUSLY MENTIONED, SKIP TO Q48E.** | 47E. Is [SERVICE] provided to a recent delivery?  (Yes=1, No=2) **ONLY** **FOR ITEMS CODED 1 IN THIS COLUMN, ASK Q48E.** | 48E. Generally who provides the [SERVICE]? **PUT √ AGAINST ALL PERSONS PROVIDING THE SERVICE** | | | | | | | | | | |
|  |  |  |  |  | Doctor | | Nurse | | | | | Others (Specify) | | | |
| 51 | Lochia (Vaginal discharge after delivery) examination | |  |  |  | | | | | | | | | | |
| 52 | Identification and management of danger signs of late postpartum complications | |  |  |  | | | | | | | | | | |
| 53 | Contraceptive/family planning counseling | |  |  |  | | | | | | | | | | |
| 54 | Counseling on danger signs in neonate & when to seek care | |  |  |  | | | | | | | | | | |
| 55 | Counseling on infant immunization | |  |  |  | | | | | | | | | | |
| 56 | Counseling on late postpartum complication & when to seek care | |  |  |  | | | | | | | | | | |
| 57 | Provision of contraceptives | |  |  |  | | | | | | | | | | |
| 58 | Referral services | |  |  |  | | | | | | | | | | |
| POST-ABORTION SERVICES | | | 46F. Please provide a list of post-abortion services provided. **FOR EACH SERVICE SPONTANEOUSLY MENTIONED, RECORD 1. FOR ITEMS NOT MENTIONED SPONTANEOUSLY, ASK Q47F, LEAVING THE CELL BLANK. FOR SERVICES SPONTANEOUSLY MENTIONED, SKIP TO Q48F.** | 47F. Is [SERVICE] related to abortion provided?  (Yes=1, No=2) **ONLY** **FOR ITEMS CODED 1 IN THIS COLUMN, ASK Q48F.** | 48F. Generally who provides the [SERVICE]? **PUT √ AGAINST ALL PERSONS PROVIDING THE SERVICE** | | | | | | | | | | |
|  |  |  |  |  | Doctor | Nurse | | | | Others (Specify) | | | | | |
| 59 | | D & C (Dilatation & Curettage) |  |  |  |  | | | |  | | | | | |
| 60 | | MVA (Manual vacuum aspiration) |  |  |  |  | | | |  | | | | | |
| 61 | | Provision of Antibiotics |  |  |  |  | | | |  | | | | | |
| 62 | | Identification of danger signs/complications |  |  |  |  | | | |  | | | | | |
| 63 | | Contraceptive/family planning counseling |  |  |  |  | | | |  | | | | | |
| 64 | | Provision of contraceptives |  |  |  |  | | | |  | | | | | |
| 65 | | Referral services |  |  |  |  | | | |  | | | | | |
| NEO-NATAL CARE SERVICES | | | 46G. Please provide a list of neonatal care services provided. **FOR EACH SERVICE SPONTANEOUSLY MENTIONED, RECORD 1. FOR ITEMS NOT MENTIONED SPONTANEOUSLY, ASK Q47G, LEAVING THE CELL BLANK. FOR SERVICES SPONTANEOUSLY MENTIONED, SKIP TO Q48G.** | 47G. Is [SERVICE] provided to newborns?  (Yes=1, No=2) **ONLY** **FOR ITEMS CODED 1 IN THIS COLUMN, ASK Q48G.** | 48G. Generally who provides the [SERVICE]? **PUT √ AGAINST ALL PERSONS PROVIDING THE SERVICE** | | | | | | | | | | |
|  |  |  |  |  | Doctor | Nurse | | | | Others (Specify) | | | | | |
| 66 | | Clean cord care |  |  |  |  | | | |  | | | | | |
| 67 | | Drying & Warmth / Kangaroo Mother Care |  |  |  |  | | | |  | | | | | |
| 68 | | Oral & Nasal Suction |  |  |  |  | | | |  | | | | | |
| 69 | | Bag & Mask ventilation |  |  |  |  | | | |  | | | | | |
| 70 | | Chest Compressions (CPR) |  |  |  |  | | | |  | | | | | |
| 71 | | Resuscitation – Intubation & ventilation |  |  |  |  | | | |  | | | | | |
| 72 | | Weigh baby |  |  |  |  | | | |  | | | | | |
| 73 | | Measure length of baby |  |  |  |  | | | |  | | | | | |
| 74 | | Oral Polio/BCG Vaccine before discharge |  |  |  |  | | | |  | | | | | |
| 75 | | Identification of neonatal danger signs/illness |  |  |  |  | | | |  | | | | | |
| 76 | | Oxygen |  |  |  |  | | | |  | | | | | |
| 77 | | Nasogastric feeds |  |  |  |  | | | |  | | | | | |
| 78 | | IV fluids |  |  |  |  | | | |  | | | | | |
| 79 | | Emollient therapy |  |  |  |  | | | |  | | | | | |
| 80 | | IM/IV Antibiotics |  |  |  |  | | | |  | | | | | |
| 81 | | IV 10% dextrose |  |  |  |  | | | |  | | | | | |
| 82 | | Care for LBW neonates |  |  |  |  | | | |  | | | | | |
| 83 | | Care for hyperbilirubinemia |  |  |  |  | | | |  | | | | | |
| 84 | | Stabilization of ill infant (Use of IV fluids, anti seizure medication) |  |  |  |  | | | |  | | | | | |
| 85 | | Neonatal check-up on day 2 |  |  |  |  | | | |  | | | | | |
| 86 | | Neonatal check-up on day 4 |  |  |  |  | | | |  | | | | | |
| 87 | | Referral Services |  |  |  |  | | | |  | | | | | |
| CHILD HEALTH SERVICES | | | 46H. Please provide a list of child health services provided. **FOR EACH SERVICE SPONTANEOUSLY MENTIONED, RECORD 1. FOR ITEMS NOT MENTIONED SPONTANEOUSLY, ASK Q47H, LEAVING THE CELL BLANK. FOR SERVICES SPONTANEOUSLY MENTIONED, SKIP TO Q48H.** | 47H. Is [SERVICE] provided ifor children?  (Yes=1, No=2) **ONLY** **FOR ITEMS CODED 1 IN THIS COLUMN, ASK Q48H.** | 48H. Generally who provides the [SERVICE]? **PUT √ AGAINST ALL PERSONS PROVIDING THE SERVICE** | | | | | | | | | | |
|  |  |  |  |  | Doctor | Nurse | | | | Others (Specify) | | | | | |
| 88 | | Use of growth chart for weight recording |  |  |  |  | | | |  | | | | | |
| 89 | | Measure weight |  |  |  |  | | | |  | | | | | |
| 90 | | Measure height |  |  |  |  | | | |  | | | | | |
| 91 | | Child Immunization |  |  |  |  | | | |  | | | | | |
| 92 | | Screening for pneumonia |  |  |  |  | | | |  | | | | | |
| 93 | | Antibiotics for ARI |  |  |  |  | | | |  | | | | | |
| 94 | | Assessment of dehydration |  |  |  |  | | | |  | | | | | |
| 95 | | Management of shock |  |  |  |  | | | |  | | | | | |
| 96 | | Management of acute encephalitis syndromes |  |  |  |  | | | |  | | | | | |
| 97 | | Referral services |  |  |  |  | | | |  | | | | | |

**H: SERVICE STATISTICS**

| Q # | Question | Jan, 2010 | Feb, 2010 | Mar, 2010 | April 2009 to March 2010 | Base document for the indicator |
| --- | --- | --- | --- | --- | --- | --- |
| 49 | # of deliveries conducted at hospital - Normal |  |  |  |  |  |
| 50 | # of deliveries conducted at hospital - Caesarean |  |  |  |  |  |
| 51 | # of live-births in hospital |  |  |  |  |  |
| 52 | # of infant deaths in the hospital |  |  |  |  |  |
| 53 | # of maternal deaths in the hospital |  |  |  |  |  |

**THANKS FOR GIVING YOUR PRECIOUS TIME**
