# Supplementary material for: Assessment of facility readiness and provider preparedness for dealing with postpartum haemorrhage and pre-eclampsia/eclampsia in public and private health facilities of northern Karnataka, India: a cross-sectional study
Source: BMC Pregnancy Childbirth. 2014 Sep 4;14:304. doi: 10.1186/1471-2393-14-304 (PMC4161844; doi:10.1186/1471-2393-14-304)
Supplement: Supplementary file 5 — Additional file 5: TEST CASE STUDY_PE_PPH, PDF. (DOCX 32 KB) [file 12884_2014_1171_MOESM5_ESM.docx]

| ***TOOL –*** | **CASE STUDY** | CONFIDENTIAL  FOR ASSESSMENT |
| --- | --- | --- |
|  | **QUALITY ASSESSMENT – MNCH PROJECT** |  |
|  |  |  |

|  |  | | | **IDENTIFICATION** |  | | | |  | | | | |
| --- | --- | --- | --- | --- | --- | --- | --- | --- | --- | --- | --- | --- | --- |
|  |  | | |  |  | | | |  | | | | |
| SERIAL NUMBER |  | |  | ID CODE |  | |  |  |  |  |  | |  |
|  | |  | | | |  | | | | | |  | |
| TYPE OF FACILITY | | 1.DISTRICT HOSPITAL | | | | 2. TALUKA HOSPITAL | | | | | |  | |
|  |  | 3. CHC | | | | 4. PHC | | | | | |  | |
|  | | 5. PRIVATE HOSPITAL | | | | 6. **OTHER (SPECIFY)** | | | | | |  | |

| NAME OF FACILITY |  |  |  |  |
| --- | --- | --- | --- | --- |

| HEALTH CARE PROVIDER | 1.MEDICAL OFFICER/IN-CHARGE | 2. OBSTETRICIAN |  |
| --- | --- | --- | --- |
|  | 3. PAEDIATRICIAN | 4. MO (AYUSH) |  |
|  | 5 NURSE/ANM | 6. **OTHER (SPECIFY)** |  |

| BASE DOCUMENT | 1.CARD | 2. REGISTER | 3. OTHER **(SPECIFY)** |  |
| --- | --- | --- | --- | --- |

| RESULT STATUS |  | 1.COMPLETED | 2. NOT COMPLETED |  |  |
| --- | --- | --- | --- | --- | --- |
|  |  | 3.REFUSED | 4. POSTPONED |  |  |
|  |  | 5. OTHER (**SPECIFY)** |  |  |  |

|  |  | DAY | | | MONTH | | | YEAR | | | |
| --- | --- | --- | --- | --- | --- | --- | --- | --- | --- | --- | --- |
| DATE OF ASSESSMENT | |  |  |  |  |  |  | **2** | **0** | **1** | **0** |

| NAME OF INTERVIEWER |  |  |  |  |
| --- | --- | --- | --- | --- |
|  |  |  |  | |
| SIGNATURE |  |  |  | |

**READ TO THE HEALTH CARE PROVIDER:**

Given below are some commonly encountered clinical case scenarios in Maternal care. Following each case study is a set of questions about the clinical assessment, diagnosis and management of such pregnant women.

**Directions** to the investigator: Please hand out the case scenarios with the questions.

Please read out each main question one by one and ask her/him to provide the answers. When she/he answers, the investigator should mark in the answer sheet provided, whether a particular item on the answer list is mentioned by the Health Care Provider; encircle 1 for Yes and 2 for No. DO NOT READ OUT THE ANSWERS.

**CASE STUDY 1 (PIH/ PRE-ECLAMPSIA/ ECLAMPSIA)**

**ANTENATAL CARE –A**

**Scenario Part One**

Mrs. C. is brought to the antenatal care (ANC) clinic by her husband after she complained of a severe headache this morning. They had been counselled on danger signs and knew that they were to come immediately to the clinic if a severe headache was noted.

The following information is available from Mrs. C.’s antenatal record 20years old, primigravida, with 37 weeks of pregnancy with c/o headache. Previous antenatal visits uneventful.

**Q1. Given the clinical information, what would you assess immediately**.

| **QN** | **QUESTIONS AND FILTERS** | **CODING** | **SKIP TO** |
| --- | --- | --- | --- |
|  | **ACTIVITIES** |  |  |
|  | **TAKE HISTORY ABOUT** | **YES NO** |  |
| 101 | Time of onset of presenting symptoms | 1 2 |  |
| 102 | Any history of convulsions in this pregnancy | 1 2 |  |
| 103 | Presence of foetal movement | 1 2 |  |
|  | **DO GENERAL EXAMINATION TO CHECK FOR** | **YES NO** |  |
| 104 | Vital signs (BP) | 1 2 |  |
| 105 | Level of consciousness | 1 2 |  |
| 106 | Abdominal tenderness | 1 2 |  |
|  | **DO EXAMINATION FOR OBSTETRIC CONDITION TO CHECK** | **YES NO** |  |
| 107 | Fundal height | 1 2 |  |
| 108 | Foetal heart rate | 1 2 |  |
| 109 | Vaginal bleeding | 1 2 |  |
| 110 | Leaking of fluid per vagina | 1 2 |  |
|  | **DO THE FOLLOWIN INVESTIGATION** | **YES NO** |  |
| 111 | Urine for protein | 1 2 |  |

**SCENARIO PART TWO**

Mrs. C. has the following findings

c/o onset of severe headache and blurred vision 6 hours before coming to the clinic.

No H/o upper abdominal pain or decreased urine output,

Foetal movement is normal.

| **ON EXAMINATION:**   - **BP 160/110 mm HG** - **Pulse 84/minute** - **Temperature 37.2°C** - **Respirations 18/minute** |
| --- |
| **PER ABDOMEN:**   - **Abdomen Non-tender** - **Fundal Height Appropriate for gestational age** - **Foetal Heart Rate 140 beats per minute** - **Contractions Two in ten minutes lasting 20 seconds by palpation** - **Patellar reflexes Normal** |
| **URINE TEST:**   - **Urine 3+protein** |

**Q2. Given the information presented above, what is your working diagnosis?**

| **QN** | **QUESTIONS AND FILTERS** | **CODING** | **SKIP TO** |
| --- | --- | --- | --- |
|  | **DIAGNOSIS** |  |  |
| 201 | Pre eclampsia/Pregnancy Induced Hypertension | YES 1  NO 2 |  |

**Q 3. What are the most urgent steps to manage this condition?**

| **QN** | **QUESTIONS AND FILTERS** | **CODING** | **SKIP TO** |
| --- | --- | --- | --- |
|  | **MANAGEMENT** | YES NO |  |
| 202 | Give Magnesium Sulphate | 1 2 |  |
| 203 | Give Anti-hypertensives | 1 2 |  |
| 204 | Immediately refer Mrs. C to a higher facility | 1 2 |  |

**ANTENATAL CARE –B**

Mrs. C. is brought to the emergency department by her husband as she was having convulsions at home. He states that she had c/o severe headache and blurred vision.

Mrs. C. has the following findings

20years old, primigravida, with 37 weeks of pregnancy with c/o headache. Previous antenatal visits uneventful.

H/o Convulsion at home

| **ON EXAMINATION:**   - **BP 160/110 mm HG** - **Pulse 84/minute** - **Temperature 37.2°C** - **Respirations 18/minute** |
| --- |
| **PER ABDOMEN:**   - **Abdomen Non-tender** - **Fundal Height Appropriate for gestational age** - **Foetal Heart Rate 140 beats per minute** - **Contractions Two in ten minutes lasting 20 seconds by palpation** - **Patellar reflexes Normal** |
| **URINE TEST:**   - **Urine 3+protein** |

**Q1. Given the information presented above, what is your working diagnosis?**

| **QN** | **QUESTIONS AND FILTERS** | **CODING** | **SKIP TO** |
| --- | --- | --- | --- |
|  | **DIAGNOSIS** |  |  |
| 301 | Eclampsia | YES 1  NO 2 |  |

**Q2.** **What are the most urgent steps to manage this condition?**

| **QN** | **QUESTIONS AND FILTERS** | **CODING** | **SKIP TO** |
| --- | --- | --- | --- |
|  | **MANAGEMENT** | YES NO |  |
| 302 | Strict bed rest | 1 2 |  |
| 303 | Give Magnesium Sulphate | 1 2 |  |
| 304 | Give Anti-hypertensives | 1 2 |  |

**Q3. If Mrs. C. had been having a convulsion at the time she came to the clinic, what IMMEDIATE actions SHOULD be taken?**

| **QN** | **QUESTIONS AND FILTERS** | **CODING** | **SKIP TO** |
| --- | --- | --- | --- |
|  | **MANAGEMENT** | **YES NO** |  |
| 305 | Administer oxygen | 1 2 |  |
| 306 | Give Magnesium Sulphate | 1 2 |  |
| 307 | Give Anti-hypertensives | 1 2 |  |
| 308 | Put patient in side-lying or lateral position | 1 2 |  |

**Q 4. What are the Essential equipments and Supplies required to manage this condition**

| **QN** | **QUESTIONS AND FILTERS** | **CODING** | **SKIP TO** |
| --- | --- | --- | --- |
|  | **EQUIPMENT AND SUPPLIES** | **YES NO** |  |
| 309 | IV with Normal Saline or Ringers Lactate | 1 2 |  |
| 310 | Indwelling urinary catheter and urinary bag | 1 2 |  |
| 311 | Suction apparatus & suction catheter | 1 2 |  |
| 312 | Oxygen & adult mask | 1 2 |  |
| 313 | Magnesium sulphate for injection | 1 2 |  |

**Scenario Part 2**

One hour following the initiation of treatment, Mrs. C. still has a moderate headache, but she has had no further convulsions.

| **ON EXAMINATION**   - BP 140/100 mmHg - Pulse 84/minute - Temp 37.2 °C - Respirations 18/minute - Chest Clear - Patellar reflexes Normal |
| --- |
| **PER ABDOMEN:**   - Abdomen Non-tender - Foetus Cephalic presentation, head not palpable above the symphysis pubis - Foetal Heart Tones 130-140 beats per minute - Contractions Three in ten minutes lasting 40-60 seconds by palpation - On P/V , Cervix Soft, 4cm dilation |
| **URINE TEST:**   - **Urine NORMAL** |

**Q5.** **What are the important steps to manage this condition?**

| **QN** | **QUESTIONS AND FILTERS** | **CODING** | **SKIP TO** |
| --- | --- | --- | --- |
|  | **MANAGEMENT** | **YES NO** |  |
| 401 | Repeat dose of magnesium sulphate four hours after the last dose if respirations, reflexes and patellar reflexes are normal | 1 2 |  |
| 402 | Give Anti-hypertensives | 1 2 |  |
| 403 | Do Artificial Rupture of Membrane(ARM) and start Oxytocin | 1 2 |  |
| 404 | Maintain Intake/Output record | 1 2 |  |
| 405 | Check respiratory rate, reflexes and patellar reflexes hourly and record | 1 2 |  |

**Scenario Part 4**

Mrs C delivered after 4 hours. A female baby was born by normal vaginal delivery. There was no PPH.BP is 140/100.

**Q 6. How long would you continue Magnesium Sulphate.**

| **QN** | **QUESTIONS AND FILTERS** | **CODING** | **SKIP TO** |
| --- | --- | --- | --- |
|  | **MANAGEMENT** | **YES NO** |  |
| 406 | Continue magnesium sulphate for 24 hours after birth under careful observation | 1 2 |  |

**CASE STUDY 3 (POSTPARTUM HAEMORRHAGE)**

**Scenario Part 1**

Mrs. B is a 30 year old gravid 4, para 4. She gave birth at the health centre to a healthy, full term baby weighing 2.6 kg. You gave oxytocin/misoprostol following the birth of the baby. The placenta was delivered 5 minutes later without complication.

However, 30 minutes after childbirth, Mrs B is having heavy vaginal bleeding.

| **QN** | **QUESTIONS AND FILTERS** | **CODING** | **SKIP TO** |
| --- | --- | --- | --- |
|  | **ACTIVITY/**  **What is the first action you will take?** |  |  |
| 601 | Check the uterus to see whether it is contracted | YES 1  NO 2 |  |
| 602 | **LIST THE MOST COMMON CAUSES OF POSTPARTUM HAEMORRHAGE** | **YES NO** |  |
| A | Uterine __atony | **1 2** |  |
| B | Retained placenta_/ placental tissue | **1 2** |  |
| C | Vaginal or cervical tears | **1 2** |  |
| D | Rupture__uterus | **1 2** |  |
| E | Bleeding disorders | **1 2** |  |
|  | **EARLY POSTPARTUM HAEMORRHAGE IS DEFINED AS**: |  |  |
| 603 | Bleeding within the first 24 hours of delivery of great than or equal to 500 ml of blood (1000ml for LSCS) | YES 1  NO 2 |  |
|  | **Vaginal bleeding immediately after birth in presence of a well contracted uterus is most often due to:** |  |  |
| 604 | Genital trauma | YES 1  NO 2 | ` |

**Scenario Part 2**

You have completed your assessment of Mrs B and your main findings are:

Pulse 88/minute, respiration rate 18/minute, BO 100/80, temperature 37 C.

Per Abdomen : Her uterus is firm and well contracted. The placenta is complete. She has no perineal trauma.

It is difficult to examine the vagina and cervix because she continues to have heavy vaginal bleeding.

| **QN** | **QUESTIONS AND FILTERS** | **CODING** | **SKIP TO** |
| --- | --- | --- | --- |
|  | **Based on these findings, what is your next step?** |  |  |
| 605 | Perform speculum examination of the vagina and cervix to identify and repair tears | YES 1  NO 2 |  |
| 606 | **What will you tell your assistant to do while you examine the patient?** | **YES NO** |  |
| A | Monitor vital signs | **1 2** |  |
| B | Begin intravenous fluids | **1 2** |  |
| C | Reassure Mrs B and her family | **1 2** |  |
| D | Draw blood for haemoglobin | **1 2** |  |

**Scenario Part 3**

One hour following childbirth you repair Mrs. B’s cervical tear. Her haemoglobin is found to be 10g/dL and her vital signs are stable.

| **QN** | **QUESTIONS AND FILTERS** | **CODING** | **SKIP TO** |
| --- | --- | --- | --- |
| 607 | **What is the appropriate plan of care?** | **YES NO** |  |
| A | Monitor her vital signs | **1 2** |  |
| B | Encourage breast feeding | **1 2** |  |
| C | Begin IFA supplementation | **1 2** |  |
